# Did the English strategy reduce inequalities in health? A difference-in-difference analysis comparing England with three other European countries

**DOI:** 10.1186/s12889-016-3505-z

**Published:** 2016-08-24

**Authors:** Yannan Hu, Frank J. van Lenthe, Ken Judge, Eero Lahelma, Giuseppe Costa, Rianne de Gelder, Johan P. Mackenbach

**Affiliations:** 1 Department of Public Health, Erasmus University Medical Centre, P.O. Box 2040, 3000 CA Rotterdam, The Netherlands; 2 Department of Health, University of Bath, Bath, UK; 3 Department of Public Health, University of Helsinki, Helsinki, Finland; 4 Department of Clinical and Biological Science, University of Turin, Turin, Italy

**Keywords:** Health inequality, English strategy, Self-assessed health, Long-standing health problems, Obesity, Smoking, Difference-in-difference analysis, Europe

## Abstract

**Background:**

Between 1997 and 2010, the English government pursued an ambitious programme to reduce health inequalities, the explicit and sustained commitment of which was historically and internationally unique. Previous evaluations have produced mixed results. None of these evaluations have, however, compared the trends in health inequalities within England with those in other European countries. We carried out an innovative analysis to assess whether changes in trends in health inequalities observed in England after the implementation of its programme, have been more favourable than those in other countries without such a programme.

**Methods:**

Data were obtained from nationally representative surveys carried out in England, Finland, the Netherlands and Italy for years around 1990, 2000 and 2010. A modified difference-in-difference approach was used to assess whether trends in health inequalities in 2000–2010 were more favourable as compared to the period 1990–2000 in England, and the changes in trends in inequalities after 2000 in England were then compared to those in the three comparison countries. Health outcomes were self-assessed health, long-standing health problems, smoking status and obesity. Education was used as indicator of socioeconomic position.

**Results:**

After the implementation of the English strategy, more favourable trends in some health indicators were observed among low-educated people, but trends in health inequalities in 2000–2010 in England were not more favourable than those observed in the period 1990–2000. For most health indicators, changes in trends of health inequalities after 2000 in England were also not significantly different from those seen in the other countries.

**Conclusions:**

In this rigorous analysis comparing trends in health inequalities in England both over time and between countries, we could not detect a favourable effect of the English strategy. Our analysis illustrates the usefulness of a modified difference-in-difference approach for assessing the impact of policies on population-level health inequalities.

**Electronic supplementary material:**

The online version of this article (doi:10.1186/s12889-016-3505-z) contains supplementary material, which is available to authorized users.

## Background

Between 1997 and 2010, the English government made reducing health inequalities part of its core political programme [27]. It developed and implemented a strategy that—in the government’s own words—was “the most comprehensive programme of work to tackle health inequalities ever undertaken in this country” [16]. This contained a number of comprehensive and coordinated policies, which were clearly documented and monitored in a series of reports [15–21, 28].

The English strategy to reduce health inequalities was shaped in two steps [40, 41], of which the first was taken in 1999, when the Department of Health issued “Reducing Health Inequalities: an Action Report” [16]. This set out national actions across a broad front including raising living standards and tackling low income, family support policies, tax-reduction and long-term care for the elderly, anti-smoking policies, improving early education (“Sure Start”) and promoting healthy communities, as well as some broader policies in the areas of education, employment and housing. It largely followed the recommendations of the Acheson committee which were based on the best available evidence in the late 1990s [15].

The second step followed in 2003 when a more focused strategy was laid down in “Tackling Health Inequalities: a Program for Action” [18]. Following an interdepartmental review of progress [17], it announced a revised strategy which contained 12 “headline indicators” (i.e., specific targets for intermediate outcomes) and 82 “departmental commitments”, that together were expected to ensure the timely delivery of two new overall targets: “to narrow the gap in life expectancy between areas and the difference in infant mortality across social classes by 10 % in 2010”. The revised strategy also had a stronger emphasis on “downstream” policies than the 1999 Action Report, such as reducing smoking in manual social groups, managing other risks for coronary heart disease and cancer (e.g., poor diet and obesity, physical inactivity, hypertension), improving housing quality by tackling cold and dampness, and reducing accidents at home and on the road. The total budget exceeded £20 billion [18].

A remarkable series of reports systematically assessing and reviewing progress in achieving “headline indicators” and fulfilling “departmental commitments” followed. The high level of government commitment to reducing health inequalities was matched by an equally remarkable commitment to critically review, revise and then re-review its policies [40]. It has been noted that, quite clearly, the English strategy to reduce health inequalities was both historically and internationally unique [39, 44].

When the strategy came to an end, however, after the election in 2010 of a new government, the results turned out to be less encouraging than most people had expected. On the one hand, all the departmental commitments were fulfilled, indicating that all elements of the strategy as originally planned had been implemented, from “Sure Start” to the creation of sports facilities, from neighbourhood renewal programmes to smoking cessation support, and from improving access to health care services to reducing fuel poverty [19, 20, 41]. This by itself was a great achievement, but only some of the headline indicators showed reduced inequalities, in terms of smaller relative or absolute inequalities in intermediate outcomes like educational outcomes, child poverty or cardiovascular risks. Others, including those that matter for inequalities in life expectancy and infant mortality, such as on primary care, diet and smoking, suggested stable or even increased inequalities between socioeconomic groups [19, 20, 41, 42]. There was no evidence at all for a reduction of inequalities in infant mortality or life expectancy, as stipulated in the overall targets [21, 41].

However, one potential problem with most existing evaluations of the English strategy is that these mainly investigated the trends in health inequalities within England after the implementation of the strategy, sometimes as compared to the trends before the implementation of the strategy, but never as compared to the trends in other countries. Given that a widening of health inequalities over the past decades has been observed in many European countries [45, 46], a relevant question is whether health inequalities in England have perhaps widened less than elsewhere thanks to the English strategy, as compared to the trends in other countries which have taken less action to tackle health inequalities.

This paper therefore extends existing evaluations by first assessing the change in trend in health inequalities in England between 1990 and 2000 (during which the English strategy had not yet been generally implemented) and 2000–2010 (during which the main effects of the strategy could be expected), and then comparing this change in trend, if any, with the trend change occurring in 3 comparison countries.

For comparison we selected countries that were in a similar stage of awareness of health inequalities, but that had not implemented a national strategy to tackle health inequalities. Our selection of countries was guided by several studies that have characterized national policy developments in this area in European countries [35, 44, 59]. Based on a strong tradition of measuring and investigating health inequalities, Finland launched a national public health programme with explicit priorities for reducing health inequalities, which were elaborated in a specific programme on reducing health inequalities conducted between 2008 and 2011. No resources however, were allocated for the latter programme except for doing more research, and it was not implemented in practice [53]). In terms of Whitehead’s action spectrum, the Netherlands was already in a phase of “structured development” in the late 1990s [59]. After 2 5-year research programmes, a national programme to tackle health inequalities was proposed in 2001, but was never implemented, mainly because of a sudden change in government [49, 50]. In Italy, a country in a stage of “concern” with regard to health inequalities according to Whitehead’s action spectrum, a serious level of awareness was evidenced by national research programmes, but again, no coordinated action to tackle health inequalities was taken by the national government [35, 44]). We did not choose other parts of the United Kingdom (Scotland, Wales and Northern Ireland) as the comparison countries because, although they may be more comparable to England, they also underwent significant policy changes to reduce health inequalities after 2000 [35].

For our evaluation of the English strategy, we used individual-level data from national health interview surveys, which allowed us to study trends in inequalities in health (self-assessed health, long-standing health problems) and in determinants of health (smoking and obesity). Like mortality and life expectancy, self-assessed health and long-standing health problems are generic health outcomes for which socioeconomic inequalities have been extensively documented [23, 37]. Many elements of the English strategy may have contributed to a favourable change in the trend in inequalities for these health outcomes, including improvements in material living conditions, health-related behaviours like smoking, diet and exercise, and access and quality of care [16, 18]. Smoking and obesity were directly targeted by the English strategy, which explicitly aimed to reduce inequalities in smoking (e.g., by increasing access to smoking cessation services) and to improve diet and physical activity [18].

We aimed to investigate the effect of the English strategy by assessing whether trends in inequalities in these health outcomes were more favourable in 2000–2010 as compared to those in 1990–2000 in England, and whether the changes in trends in inequalities after 2000 in England were more favourable than those in the three comparison countries.

## Methods

### Data

We obtained nationally representative health surveys or multipurpose surveys with a health component from England, Finland, the Netherlands and Italy (Table 1). The available years of surveys differed slightly between countries, but all of them were around 1990, 2000 or 2010. The selected surveys were identical over time for England and Finland, but not for the Netherlands and Italy. Given that our main aim was to investigate the changes in trends in health inequalities between 1990–2000 and 2000–2010 in England and whether these changes in trends were more favourable in England than in the three comparison countries, we focused on the comparability over time within each country and considered the risk of bias due to between-country variations in data collection to be limited. All the selected surveys had a high degree of comparability within-country over time in the aspects of sampling strategy, survey questions and answers, thus could be used to analyse the trends over time [11, 24, 31–33, 36, 55]. Details on data collection in each country are reported in the Additional file 1: Table SA1. The age range used in the analyses was 16–79 years. Older respondents were excluded to avoid the potential bias caused by the exclusion of institutionalized population in most surveys. Survey weights which were designed to make the sample representative of the whole population were available in some countries and years. Specifically, weights were available for the data from England in 2010, the Netherlands in 1990/2000/2010 and Italy in 1990/2000. Most of the weights are normal sampling weights, which make the samples nationally representative. Weights in the Dutch survey in 2000 and 2010 are “expansion” weights, which are used so that the weighted data reflect the size of the total Dutch population. In order to be comparable to those in the other years and other countries, weights in the Dutch survey in 2000 and 2010 were scaled in our analysis (i.e. divide each year by the mean weight).

**Table 1:** Countries included in the analysis and sources of data

Country	Survey year	Survey names
England	1991–1992; 2000; 2010	Health Survey for England
Finland	1989; 1999; 2009	Health Behaviour and Health
The Netherlands	1990	Ongoing Survey of Living Conditions (DLO)
	2000; 2009	Permanent Survey of Living Conditions (POLS)
Italy	1990	Multipurpose Family Survey
	2000	Health and Health Care Utilization
	2010	Multipurpose Family Survey-Aspects of daily living

The Finnish data used in this study are the data combined from the two Finish studies: “Health behaviour and health among Finnish adult population (AVTK)”, which includes respondents who are 15–64 years old, and “Health behaviour and health among the Finnish elderly (EVTK)”, which includes respondents who are older than 64 years

Based on data availability, four health outcomes were chosen: self-assessed health, long-standing health problems, smoking status and obesity. Self-assessed health was generated based on a question which was framed in a way similar to “how is your health in general?”, and was recoded into a binary variable indicating whether the respondent had less-than-good self-assessed health. Long-standing health problems was a binary variable measuring whether or not the respondent reported any long-standing health problems. Smoking status was measured as whether the respondent was a current smoker. Obesity was based on the body mass index (BMI) of 30 or higher, calculated from the measured or self-reported height and weight (kg/m^2^). The precise survey questions and answer categories varied slightly between countries, but consistency over time was retained in all countries (Additional file 1: Table SA1).

Socioeconomic position was measured by the highest level of education completed or currently being attended by a person. It was harmonized on the basis of the International Standard Classification of Education (ISCED) and reclassified into 3 categories: levels 0–2 (no, primary or lower secondary education, considered “low-educated”), levels 3–4 (upper secondary and post-secondary non-tertiary education, considered “middle-educated”), levels 5–6 (tertiary education, considered “high-educated”). Details in the classification of education in England are reported in the Additional file 1: Table SA2). Comparable indicators for other measures of socioeconomic position, such as occupational class or income level, were not available in all surveys.

### Statistical methods

Our analysis started with a comparison of changes in health occurring between 1990 and 2000 (control condition) and those occurring between 2000 and 2010 (treatment condition) among low-educated people in England. We assessed whether there was a larger improvement of health among low-educated people in England after the introduction of the strategy than before the introduction of the strategy.

The model for this analysis can be written as:

$$ outcom{e}_{ist}={\beta}_0+{\beta}_1 endyea{r}_t+{\beta}_2 policyperio{d}_s+{\beta}_3 endyea{r}_t* policyperio{d}_s+{X}_{ist} $$

where *outcome*_*ist*_ is one of the chosen health measures of individual i in period s and year t, *β*_0_ is a constant, *endyear*_*t*_ is a dummy indicating whether it is the end year of each period, *policyperiod*_*s*_ is a dummy indicating whether it is the period 2000–2010 (treatment period), *endyear*_t_*policyperiod_*s*_ is the interaction between *endyear*_*t*_ and *policyperiod*_*s*_, *X*_*ist*_ represents the control variables which are age and sex. For the period 1990–2000, *policyperiod*_*s*_ was 0, and *endyear*_*t*_ was 0 for data from 1990 and was 1 for data from 2000. For the period 2000–2010, *policyperiod*_*s*_ was 1, and *endyear*_*t*_ was 0 for data from 2000 and was 1 for data from 2010. The *β*_1_ coefficient measures the trend in health in the control condition (i.e. trend in 1990–2000). *β*_2_ measures the difference in the level of health between the control and treatment condition at the beginning (i.e. difference in health between the year 1990 (the beginning year of the control condition) and the year 2000 (the beginning year of the treatment condition)). *β*_3_ is the key parameter (further referred to as “two-way interaction” parameter) that quantifies the difference in the trend between the two conditions. In order to make a causal interpretation, the assumption we need is that in the absence of the strategy, the trend in health among low-educated people in 2000–2010 (treatment condition) would have been the same as the trend in health in 1990–2000 (control condition).

In a second step, in order to assess whether there is a more favourable trend in health inequalities after the introduction of the strategy, we made a comparison between the changes in improvement of health between low- and high-educated people. Therefore, we introduced an additional difference, i.e. the difference between low- and high-educated people, into the regression, by adding the binary variable for education, and all possible interactions with education in the equation.

The model can be written as:

$$ \begin{array}{l} outcom{e}_{ist}={\beta}_0+{\beta}_1 endyea{r}_t+{\beta}_2 policyperio{d}_s+{\beta}_3 endyea{r}_t* policyperio{d}_s+{\beta}_4 led{u}_{ist}\\ {}\kern3.48em +{\beta}_5 led{u}_{ist}* endyea{r}_t+{\beta}_6 led{u}_{ist}* policyperio{d}_s+{\beta}_7 led{u}_{ist}* endyea{r}_t\\ {}\kern3.48em * policyperio{d}_s+{X}_{ist}\end{array} $$

where a new variable *ledu*_*ist*_ indicating whether the respondent is low-educated, and the interactions between *ledu*_*ist*_ and other variables were added. Now *β*_7_ is the key parameter (further referred to as “three-way interaction” parameter), which quantifies the difference between low and high educated in the difference in the trend between the two periods. In other words, this assessed whether the trend in health inequalities in England was different in the period 2000–2010 as compared to the period 1990–2000. In order to interpret this as the effect of the strategy, the assumption we need to make is that, in the absence of the strategy, the trend in health inequalities in both periods would have been the same. This model was also applied, independently, to the three comparison countries.

In the last and our main step, we added each of the comparison countries separately to the analysis of the English data, following the idea of “difference-in-differences analysis” [2, 60]. Our aim was to investigate whether the changes in trends in health inequalities between 1990–2000 and 2000–2010 were more favourable in England than those in the three comparison countries. The rationale, as mentioned in the introduction, is that even if there is no more reduction in health inequalities after the implementation of the strategy than before, the changes in trends in England could still be more favourable than those in other European countries that have done less to reduce health inequalities. Therefore, we pooled data from England and each of the comparison countries, and added an additional difference, i.e. the difference between England and the comparison country, into the regression, by adding a dummy for England and the corresponding interactions. Here the difference in trend in health inequalities between the period 1990–2000 and 2000–2010 in the comparison country was regarded as the control condition.

The model can be written as:

$$ \begin{array}{l} outcom{e}_{istj}=\Big({\beta}_0+{\beta}_1 endyea{r}_{tj}+{\beta}_2 policyperio{d}_{sj}+{\beta}_3 endyea{r}_{tj}* policyperio{d}_{sj}+{\beta}_4 led{u}_{istj}\\ {}\kern3.48em +{\beta}_5 led{u}_{istj}* endyea{r}_{tj}+{\beta}_6 led{u}_{istj}* policyperio{d}_{sj}+{\beta}_7 led{u}_{istj}* endyea{r}_{tj}\\ {}\kern3.48em * policyperio{d}_{sj}+{X}_{istj}\left)+\right({\beta}_0^{\mathit{\hbox{'}}}+{\beta}_1^{\mathit{\hbox{'}}} endyea{r}_{tj}+{\beta}_2^{\mathit{\hbox{'}}} policyperio{d}_{sj}+{\beta}_3^{\mathit{\hbox{'}}} endyea{r}_{tj}\\ {}\kern3.48em * policyperio{d}_{sj}+{\beta}_4^{\mathit{\hbox{'}}} led{u}_{istj}+{\beta}_5^{\mathit{\hbox{'}}} led{u}_{istj}* endyea{r}_{tj}+{\beta}_6^{\mathit{\hbox{'}}} led{u}_{istj}\\ {}\kern3.48em * policyperio{d}_{sj}+{\beta}_7^{\mathit{\hbox{'}}} led{u}_{istj}* endyea{r}_{tj}* policyperio{d}_{sj}+{X}_{istj}\Big)* englan{d}_j\end{array} $$

where *outcome*_*istj*_ is one of the chosen health measures of individual i in period s, year t and country j. Now *β*_7_^'^, the coefficient of the quadruple interaction term “*ledu*_istj_*endyear_tj_*policyperiod_sj_*england_*j*_”, is the key parameter of this model (further referred to as “four-way interaction” parameter), which quantifies the difference between the “three-way interaction” parameter of England and that of each comparison country. In other words, it assesses whether changes in trends in health inequalities observed in England after the implementation of its programme, were more favourable than those in other countries without such a programme. In order to make a causal interpretation, the assumption is that in the absence of the strategy, the changes in trends in health inequalities between the two periods in England would have been the same as those in the comparison countries.

Logistic regression was used in all the analyses. When the outcomes are non-linear, as in the case of binary outcomes, difference-in-difference models for non-linear models (such as logistic regression) are to be preferred [54]. The interpretation of the interaction terms in difference-in-differences logistic models is essentially similar to that in the more common linear models, except that they indicate the relative change of the odds of the health outcome in the treatment group relative to that in the control group, instead of the absolute change of the rate of the health outcome in the treatment group minus that in the control group. Robust standard errors were used to account for potential heteroskedasticity. Unweighted results are reported in the results section. Analysis with weighting factors when available are reported in the appendix as a sensitivity analysis (Additional file 1: Table SA3).

All regression analyses were performed in Stata 13.1. Results with a *p*-value lower than 0.1 were regarded as significant. The specific significance level was indicated for each significant result. The coding of the variables and more explanations are reported in the appendix.

## Results

Summary statistics of key variables are presented in Table 2. Compared to the three comparison countries, the sample in England appeared to have a more equal distribution of the three education categories, a relatively lower proportion of less-than-good self-assessed health, a higher proportion of long-standing health problems, an average level of smoking prevalence, but a much higher rate of obesity.

**Table 2:** Summary statistics of key variables, pooled for all years in each country

	England		Finland		The Netherlands		Italy	
	*N*	%	*N*	%	*N*	%	*N*	%
Number of respondents	22,442		14,296		18,353		204,963	
Gender								
Male	10,255	46 %	6654	47 %	8712	47 %	99,888	49 %
Female	12,187	54 %	7642	53 %	9641	53 %	105,075	51 %
Age								
16-25	3057	14 %	1874	13 %	2816	15 %	32,779	16 %
26–35	4102	18 %	1959	14 %	3484	19 %	37,242	18 %
36–45	4366	19 %	2208	15 %	3627	20 %	37,626	18 %
46–55	3787	17 %	2181	15 %	3179	17 %	34,499	17 %
56–65	3403	15 %	2096	15 %	2648	15 %	30,930	15 %
66–79	3727	17 %	3978	28 %	2599	14 %	31,887	16 %
Education								
ISCED 0–2	7796	36 %	4277	34 %	7880	43 %	125,976	61 %
ISCED 3–4	8127	37 %	6037	48 %	6538	36 %	64,068	31 %
ISCED 5–6	5864	27 %	2179	18 %	3877	21 %	14,919	8 %
missing	655	3 %	1803	13 %	58	0 %	0	0 %
Self-assessed health								
Less-than-good	5311	24 %	5668	40 %	4155	23 %	–	–
Good or above	17,115	76 %	8522	60 %	14,197	77 %	–	–
Missing	16	0 %	106	0 %	1	0 %	–	–
Long-standing health problems								
Yes	9338	42 %	–	–	6298	34 %	–	–
No	13,094	58 %	–	–	12,050	66 %	–	–
Missing	10	0 %	–	–	5	0 %	–	–
Smoking status								
Current smoker	5812	26 %	3409	25 %	5154	33 %	52,622	26 %
Ex or never smoker	16,527	74 %	10,380	75 %	10,571	67 %	151,675	74 %
Missing	103	0 %	507	3 %	2628	14 %	666	0 %
Obesity								
Yes	4087	21 %	1778	13 %	–	–	17,266	9 %
No	15,568	79 %	12,238	87 %	–	–	181,925	91 %
Missing	2787	12 %	280	2 %	–	–	5772	3 %

The population distribution for each variable is given as % of subjects, excluding those with missing information. The % missing for each variable is given as a % of total subjects

**Fig. 1:**
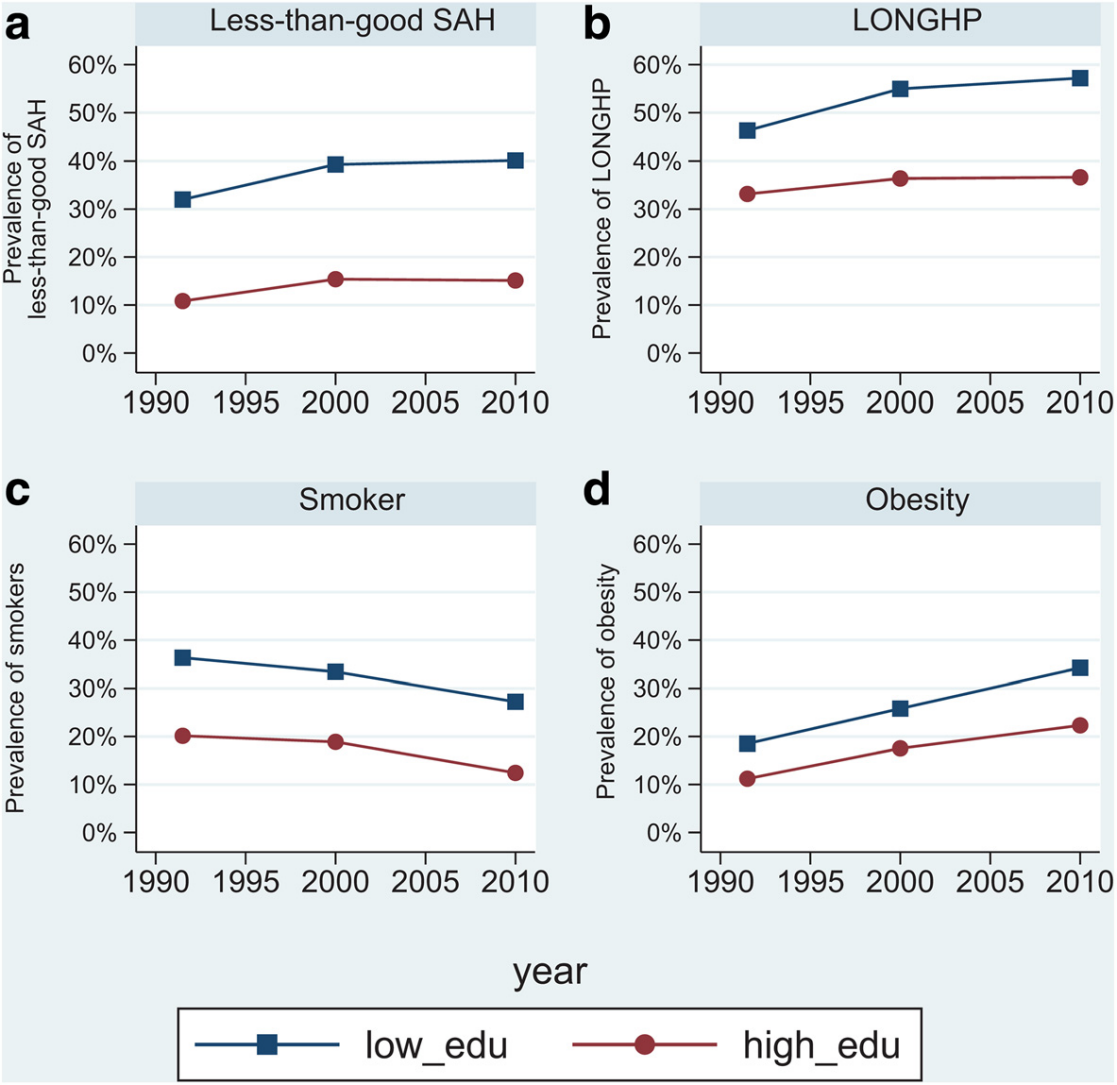
Trends in self-reported health outcomes in England by education

The main results are reported in Table 3. The full model results are reported in the appendix. The “two-way interaction” parameter estimates for low-educated people in England show that more favourable trends after 2000 were found in all health measures, although not statistically significant for obesity. A favourable change in trend is shown by odds ratios (OR) that are smaller than 1. For example, although the odds of less-than-good self-assessed health increased during both periods (Fig. 1a), the increase of the odds in 2000–2010 was 24 % less than that in 1990–2000 (OR = 0.76, *p* < 0.01). Similarly, although the odds of being a current smokers decreased during both periods (Fig. 1c), the decrease of the odds in 2000–2010 was 18 % more than that in 1990–2000 (OR = 0.82, *p* < 0.05).

**Table 3:** “Two-way interaction” parameter estimates comparing the trends in health between 1990s and 2000s, “three-way interaction” parameter estimates comparing the trends in health inequalities between 1990s and 2000s, and “four-way interaction” parameter estimates comparing the “three-way interaction” parameter estimates between countries

	Odds ratios (logistic)			
	Less-than-good self-assessed health	Long-standing health problems	Smoker	Obesity
1. Two-way interaction parameter estimates^a^				
England (low-edu)	0.76***	0.78***	0.82**	0.97
	(0.064)	(0.065)	(0.073)	(0.097)
2. Three-way interaction parameter estimates^b^				
England	1.22	0.95	1.19	1.25
	(0.197)	(0.125)	(0.182)	(0.213)
Finland	0.78	–	1.28	1.90*
	(0.173)	–	(0.308)	(0.652)
The Netherlands	1.18	1.16	1.00	–
	(0.221)	(0.181)	(0.165)	–
Italy	–	–	0.97	0.76*
	–	–	(0.072)	(0.121)
3. Four-way interaction parameter estimates^c^				
England vs Finland	1.57	–	0.93	0.66
	(0.433)	–	(0.267)	(0.253)
England vs the Netherlands	1.04	0.82	1.20	–
	(0.257)	(0.167)	(0.270)	–
England vs Italy	–	–	1.23	1.64**
	–	–	(0.209)	(0.383)

Robust standard errors in parentheses. *** *p* < 0.01, ** *p* < 0.05, * *p* < 0.1

^a^Based on the “two-way interaction” analysis for low-educated people in England. An odds ratio below 1.00 indicates a larger health improvement in the period 2000–2010 than in the period 1990–2000

^b^Based on the “three-way interaction” analysis within each country. An odds ratio below 1.00 indicates a more favourable trend in health inequalities in the period 2000–2010 than in the period 1990–2000

^c^Based on the “four-way interaction” analysis for England and each of the comparison countries. An odds ratio below 1.0 indicates a more favourable change (between 1990–2000 and 2000–2010) in the trend in health inequalities in England as compared to the other country

Table 3 also presents the “three-way interaction” parameter estimates for each country, which tested the differences in trends in health inequalities between 1990–2000 and 2000–2010. An odds ratio below 1.00 indicates that the trend in health inequalities was more favourable in the 2000s (i.e., less increase or more decrease). All three-way interactions were statistically non-significant in England, implying that trends in health inequalities after 2000 did not significantly differ from those observed in the 1990s. As shown in Fig. 1, this is because high-educated people also experienced favourable changes in trends after 2000. For the Netherlands, all three-way interactions were also statistically non-significant. Finland showed a significantly less favourable trend in inequalities in obesity after 2000 (OR = 1.90, *p* < 0.1). A more favourable change in trends of inequalities in obesity after 2000 was found in Italy (OR = 0.76, *p* < 0.1).

The results of the “four-way interaction” parameter estimates are reported in the last part of Table 3, which tested the differences in the “three-way interaction” parameter estimates between England and the comparison countries. An odds ratio below 1.00 indicates that the change in trends of inequalities was more favourable in England than in the comparison countries (i.e., a stronger change towards decreasing inequalities, or a weaker change towards increasing inequalities). Results showed that changes in trends of inequalities after 2000 were not statistically significantly different between England and any of the other countries, with the single exception of obesity for which the change was less favourable in England than in Italy (OR = 1.64, *p* < 0.05).

Using the amount of smoking per day among the current smokers as the health outcome did not change our conclusions (reported in the appendix, together with the full model results of the other outcomes). Essentially similar results were obtained in a sensitivity analysis (Additional file 1: Table SA3), where weighting factors were incorporated when available. Trends in the health outcomes in the comparison countries during the study period are also reported in the Additional files 2, 3 and 4: Figure SA1, SA2, and SA3. Additionally adding age square and the interaction between age and sex in the regressions to account for a potential nonlinear or sex-specific effect of age on health did not essentially change our results (available upon request).

## Discussion

### Summary of findings

After the implementation of the English strategy, more favourable trends in some health indicators were observed among low-educated people, but trends in health inequalities in 2000–2010 in England were not more favourable than those observed in the period 1990–2000. For most health indicators, changes in trends of health inequalities after 2000 in England were also not statistically significantly different from those seen in the other countries.

### Strengths and limitations

To the best of our knowledge, this is the first attempt to evaluate the population-level effects of the English strategy comparing trends in health inequalities in England before and after the implementation of the strategy, and between England and countries without a national programme to reduce health inequalities. We assessed whether there were larger improvements of health or health inequalities in England after the introduction of the strategy as compared to the pre-treatment trends, and simultaneously incorporated each of the comparison countries into the analysis to assess whether the changes in trends in health inequalities observed in England between the period 2000–2010 and the period 1990–2000 were more favourable than those in other countries without such a programme. For these analyses we developed a modified difference-in-difference analysis, based on a four-way interaction framework (education*time*policy*country) that may also be useful for the evaluation of other programs and interventions to tackle health inequalities. However, our study also has a number of potential weaknesses that need to be taken into account in interpreting our findings.

Our positive findings of changes in trends for the low educated in England will be biased if in the absence of the strategy, trends in health among the low educated would also have been more favourable in the second than in the first period. Potential reasons could include changes in major background causes of ill health not targeted by the strategy, resulting in, for example, less unemployment or higher incomes among lower educated. These changes should then be unique to England, since more favourable trends in health among the low educated in the period 2000–2010 were not generally observed in the comparison countries (Additional file 1: Table SA4). We consider this unlikely, and therefore think that the more favourable changes in trends among the low educated in England can be interpreted as possible effects of the English strategy on health outcomes in this group.

Analogously, our finding of an absence of significant differences in trends in inequalities before and after the implementation of the strategy in England will be biased if in the absence of the strategy, trends in health inequalities would have been less favourable in the second than in the first period. One possible candidate for a background factor which may have increased health inequalities is the Great Recession that started in the late 2000s, which may have been especially harmful for the health of vulnerable populations and may have increased health inequalities [4, 14, 57]. However, there are two reasons why we believe the resulting bias can only be very limited. First, our last measure is for 2010, i.e. shortly after the recession started, and several of our health measures (e.g. long-standing health problems and obesity) are not likely to change within 1–2 years. Second, and more importantly, we compared the changes of trends in health inequalities in England to those in 3 comparison countries that also went through the recession and we found the changes in trends in England were not significantly more favourable. One remaining concern is that if the effect of the recession on health inequalities was different for each country, our results may still be biased. However, in order to mask the effect of the English strategy, England should be the country in which health inequalities were most affected by the recession. This is unlikely, given that the UK had similar or even smaller percentage decreases of GDP and employment as compared to Italy and Finland during the period of recession [9], and the UK did not show an increase in inequalities in self-reported health and some other health measures caused by the recession [3, 4]. We therefore think that the absence of more favourable changes in health inequalities in England can be interpreted as evidence for the absence of an effect of the English strategy on health inequalities. Changes in social mobility could also affect the degree of inequalities over time, but it is unclear whether a rise in social mobility would lead to wider or narrower health inequalities [7].

The validity of the comparison with the three other countries also hinges on whether trends in inequalities in health before the implementation of the English strategy were the same in England and the other countries, and whether other countries have indeed done much less than England to reduce health inequalities. Kunst et al. [37] investigated trends in socioeconomic inequalities in self-assessed health in 10 European countries between the 1980’s and 1990’s. Their analyses showed a high degree of stability of socioeconomic inequalities in self-assessed health across the 10 countries, which included England, Finland, Italy and the Netherlands. Trends in educational inequalities in self-assessed health between 1980 and 1990 appeared to be similar in all four comparison countries, with the exception of males in Italy. In our own analysis, the changes in the odds ratios of educational inequalities in self-assessed health between 1980 and 1990 also appeared to be similar in all four comparison countries, with the exception of males in Italy. A possible improvement on our approach is the creation of a weighted “synthetic” control group, including several comparison countries [1]. In this innovative approach, weights are calculated such that the resulting synthetic control group best reproduces the values of a set of predictors of health inequalities in England before the implementation of the English strategy. We recommend to explore the usefulness of this approach for cross-national policy evaluations in future studies.

As stated in the introduction, there can be little doubt that England has done more to reduce health inequalities, but efforts to reduce health inequalities were not completely absent in the other countries. For example, by building a systematic evidence-base for interventions and policies, the Dutch government pursued a research-based approach to tackle socioeconomic inequalities in health [49]. Some of the recommendations were adopted by health policy-makers and health care practice, although more so at the local than at the national level [50]. Nevertheless, the absence of a reduction in health inequalities in the comparison countries suggests that it is unlikely that we have missed major policy effects.

Policies not specifically implemented with the purpose to reduce inequalities potentially could have influenced our findings. For example, the Dutch government increased the health care expenditure after 2001 [56]. Italy introduced a much more comprehensive smoke-free legislation in 2005, which made public transport completely smoke-free and extended coverage to bars and restaurants [13]. These policies could affect our results if their impacts were larger among lower as compared to higher socioeconomic groups. However, the extent to which these policies effectively reduce health inequalities is still rather unknown [10, 43]. Moreover, such policy changes are often specific to one country or one health outcome. Given that we have used three comparison countries and different health outcomes, and observe no significant decline of health inequalities in the comparison countries, it seems unlikely that such policies have influenced our findings substantially.

The validity of our analysis would also be compromised if the composition of the population would have changed differently in England as compared to the comparison countries. This “common composition” assumption would be violated if the UK had larger inward migration of persons with poorer or better health than the comparison countries. However, there is no evidence that changes in population composition are substantially different between these countries, as shown by statistics on the distribution of foreigners in European countries in 1980 and in 2000 [58]. Moreover, participation of migrants in surveys is usually low, so the potential for bias in our findings by immigration is also low.

Our analysis is limited by the fact that we have only used data on self-reported health measures obtained from the survey data (self-assessed health, long-standing health problems, smoking and obesity), and not on life expectancy and infant mortality, the overall targets of the English strategy. The main reason for this is the lack of comparable data for a sufficient number of countries and correct time-periods. There is evidence suggesting that inequalities in infant mortality between manual and non-manual occupational groups started to decrease in England after 2007 [5]—a possible effect of the English strategy which we have missed in our study. The only mortality outcome for which we could repeat our analyses is all-cause mortality in England and Finland (see Additional file 1: Table SA5). The analysis shows that the trend in mortality among the low educated in England was more favourable after the year 2000 than before, and that there was a smaller increase of inequalities in mortality in England after the year 2000 than before. However, the trend in inequalities in England was not statistically significantly different from that observed in Finland, which is consistent with our findings based on the survey data.

The two general health measures used in our analysis have been widely used in other comparative studies [12, 47] and have been shown to be reliable and valid indicators of general health and well-being [51]. They are also more likely to be changed in a short time span than mortality. The latter is also true for smoking, but less so for obesity. As explained in the introduction, favourable trends in inequalities in the chosen measures could reasonably be expected as a result of the English strategy, either because it directly targeted these outcomes (as in the case of smoking and obesity) or because it had more generalized effects beyond mortality (as in the case of self-assessed health and long-standing health problems). However, there is a potential concern that the implementation of the English strategy may change the willingness to report health problems of the respondents. It is therefore important to repeat our analysis with mortality and other more objective outcomes directly relevant for the strategy, if adequate data can be found.

We have used education as an indicator of socioeconomic position, which is one of the common socioeconomic indicators used in measuring health inequalities in European countries [12, 23, 37, 48]. Furthermore, education is strongly (albeit not perfectly) associated with both occupational class and income, and trends in health inequalities by education are often similar to those by occupational class or income [29]. However, the objectives of the English strategy were phrased in terms of occupational class or area-based deprivation. Comparable measures of occupational class or area-based deprivation were not available in our data. We believe that it is reasonable to assume that policies that have effectively reduced inequalities in health by occupational class or area-based deprivation, will also have reduced inequalities in health by education. To the extent however, that the effect on the first was larger than that on the second, our use of education as socioeconomic indicator may have led to underestimation of the effect of the English strategy. We therefore recommend replication of our findings in a cross-national framework with other socioeconomic indicators, if these can be found.

We adjusted for gender in all analyses, in order to increase statistical power. Because trends in health outcomes may differ between men and women, especially for smoking [26], we repeated the analyses stratified by gender (see Additional file 1: Tables SA6 and SA7). Among both men and women, for most of the health indicators changes in trends of health inequalities after 2000 in England were not statistically significantly different from those seen in the other countries. Out of 22 four-way interactions, only two showed more favourable trends in inequalities in England as compared to one of the other countries: long-standing health problems among men as compared to the Netherlands, and amount of smoking among women as compared to Italy.

In all countries, the response rate of the survey went down overtime, except in the Netherlands where it went up (Additional file 1: Table SA1). If the non-response population mainly consisted of people with low socioeconomic status or bad health, a decreasing response rate may potentially lead to more favourable trends in health inequalities. This might have biased the comparison between England and the Netherlands (but not the other two comparison countries). In our analysis, data from 3 years with 10-year gaps were used for each country. Although the choice of year is meaningful (the period of 2000–2010 is the period when the main effects of the strategy could be expected and the period of 1990–2000 is a comparable period when the strategy had not yet been generally implemented), the limited number of years implies that our measure of change may be unreliable. Further study may consider to repeat our analysis by using time-series data if available, which can help to model the trends better and improve the robustness. We mainly focused on the odds ratios of the core parameters and their statistical significance in the models, since these parameters could directly answer our study questions. In order to interpret the results better, future research may consider to present the predicted probabilities and use them to calculate meaningful results, such as the potential health or health inequalities in England in 2010 if it followed the trends of health or health inequalities in Finland. This was not done in our analysis since most of the “four-way interaction” parameters were insignificant.

### Interpretation

Taking into account the trends in inequalities before the implementation of the strategy in England and the trends in the other three European countries, we found that the effects of the English strategy on inequalities in self-assessed health, long-standing health problems, smoking and obesity were limited. Our study confirms previous evaluations which have also not found clear effects of the English strategy on the population level [19–21]. Although evaluation studies have sometimes found positive effects in specific sections of the population, e.g. small but significant reductions in the absolute and relative rate gaps in smoking prevalence between Spearhead areas and others [8], and some beneficial effects of the Sure Start Local Programmes on children and their families living in deprived communities [52], the general consensus is that population-level effects have been largely absent. A possible exception might be tackling inequalities in infant and maternal health outcomes, where a national support team was established and some positive results were reported [22].

The potential reasons for why the English strategy was not more successful have been discussed in some reviews [20, 21, 25, 28, 30, 34, 40, 41]. One widely acknowledged reason is that the design of the English strategy was not based on policies or interventions with proven effectiveness in reducing health inequalities [6, 27, 38, 40, 41]. This is partly because of the reality that the current evidence for the effectiveness of policies is limited. Another reason is that the English strategy might have chosen the wrong entry-points. Mackenbach [41] pointed out that the strategy spent resources on entry-points which were irrelevant for life expectancy or infant mortality, at least within the chosen time frame. Marmot [21] noticed that the strategy had not systematically addressed the background causes of ill health and had relied more on tackling proximal causes (such as smoking). The inadequate delivery of the English strategy was also criticized [21, 40, 41].

The detected trends in inequalities in the three comparison countries are also not generally consistent with the efforts that have been made in each country in reducing health inequalities. Finland, which has a long tradition in eliminating inequalities [35] showed less favourable trends (although many not statistically significant) in inequalities in several outcomes in recent years as compared to the 1990s. Italy, which has made less efforts to tackle health inequalities, displayed decreasing trends in inequalities in all available measures, although significant decreases could only be shown in inequalities in obesity. Similar findings are reported in the literature on trends in health inequalities in Europe, and has been attributed to the fact that Italy is relatively late in many modern epidemic transitions [45, 46]. Apparently, more effective policies together with a deeper exploration of the causes for changes in health inequalities are needed.

## Conclusions

In this rigorous analysis comparing trends in health inequalities in England both over time and between countries, we could not detect a favourable effect of the English strategy on national trends in educational inequalities in self-assessed health, long-standing health problems, smoking or obesity. However, our analysis illustrates the usefulness of a modified difference-in-difference approach for assessing the impact of policies on population-level health inequalities.

## Additional files

Additional file 1: Web appendix. (DOCX 79 kb) Additional file 2: Figure SA1. (JPG 886 kb) Additional file 3: Figure SA2. (JPG 891 kb) Additional file 4: Figure SA3. (JPG 775 kb)

## Abbreviations

BMI: body mass index
OR: odds ratio

## References

[1] Abadie A, Diamond A, Hainmueller J (2010). Synthetic control methods for comparative case studies: estimating the effect of California’s Tobacco Control Program. J Am Stat Assoc.

[2] Angrist JD, & Pischke JS. Mostly harmless econometrics. Princeton, New Jersey: Princeton University Press; 2009.

[3] Astell-Burt T, Feng X (2013). Health and the 2008 economic recession: evidence from the United Kingdom. PLoS One.

[4] Bacigalupe A, Escolar-Pujolar A (2014). The impact of economic crises on social inequalities in health: what do we know so far?. Int J Equity Health.

[5] Bambra C (2012). Reducing health inequalities: new data suggest that the English strategy was partially successful. J Epidemiol Community Health.

[6] Bambra C, Smith KE, Garthwaite K, Joyce KE, Hunter DJ (2011). A labour of Sisyphus? Public policy and health inequalities research from the Black and Acheson Reports to the Marmot Review. J Epidemiol Community Health.

[7] Bartley M, Plewis I (1997). Does health-selective mobility account for socioeconomic differences in health? Evidence from England and Wales, 1971 to 1991. J Health Soc Behav.

[8] Bauld L, Judge K, Platt S (2007). Assessing the impact of smoking cessation services on reducing health inequalities in England: observational study. Tob Control.

[9] Bell, DNF, & Blanchflower, DG. Recession and Unemployment in the OECD. 2010. CESifo Forum 1/2010, https://www.cesifo-group.de/portal/page/portal/968433588CCE0D9FE04400144FAFBA7C. (Accessed 22 July 2015).

[10] Brown T, Platt S, Amos A (2014). Equity impact of population-level interventions and policies to reduce smoking in adults: a systematic review. Drug Alcohol Depend.

[11] Carreras G, Gorini G (2014). Time Trends of Italian Former Smokers 1980–2009 and 2010–2030 Projections Using a Bayesian Age Period Cohort Model. Int J Environ Res Public Health.

[12] Cavelaars AE, Kunst AE, Geurts JJ, Crialesi R, Grotvedt L, Helmert U (1998). Differences in self reported morbidity by educational level: a comparison of 11 western European countries. J Epidemiol Community Health.

[13] Currie L, Nguyen L, Rosenqvist G, Pekurinen M (2012). Appendix A. Tobacco Control Policy Index. Demand for Tobacco in Europe: An Econometric Analysis of 11 Countries for the PPACTE Project.

[14] De Vogli R (2014). The financial crisis, health and health inequities in Europe: the need for regulations, redistribution and social protection. Int J Equity Health.

[15] Department of Health. Independent inquiry into inequalities in health (the Acheson report). London. 1998. https://www.gov.uk/government/publications/independent-inquiry-into-inequalities-in-health-report. (Accessed 16 Oct 2014).

[16] Department of Health. Reducing health inequalities: An action report. London. 1999. http://www.swslim.org.uk/downloads/SL1264.pdf. (Accessed 02 Feb 2015).

[17] Department of Health. Tackling health inequalities: Cross-cutting review. London. 2002. http://www.dh.gov.uk/prod_consum_dh/groups/dh_digitalassets/@dh/@en/documents/digitalasset/dh_4068003.pdf. (Accessed 02 Feb 2015).

[18] Department of Health. Tackling health inequalities: A program for action. London. 2003. http://webarchive.nationalarchives.gov.uk/20031220221853/doh.gov.uk/healthinequalities/programmeforaction/. (Accessed 02 Feb 2015).

[19] Department of Health. Tackling health inequalities: 2007 status report of the program for action. London. 2007. http://webarchive.nationalarchives.gov.uk/+/www.dh.gov.uk/en/Publicationsandstatistics/Publications/DH_083471. (Accessed 02 Feb 2015).

[20] Department of Health. Tackling health inequalities: 10 years on. London. 2009. http://webarchive.nationalarchives.gov.uk/20130107105354/http:/www.dh.gov.uk/prod_consum_dh/groups/dh_digitalassets/documents/digitalasset/dh_098934.pdf. (Accessed 02 Feb 2015).

[21] Department of Health. Fair society, healthy lives (the Marmot Review). London. 2010a. http://www.instituteofhealthequity.org/projects/fair-society-healthy-lives-the-marmot-review. (Accessed 02 Feb 2015).

[22] Department of Health. Tackling health inequalities in infant and maternal health outcomes (report of the infant mortality national support team). London. 2010b. https://www.gov.uk/government/uploads/system/uploads/attachment_data/file/215869/dh_122844.pdf. (Accessed 16 Apr 2015).

[23] Eikemo TA, Huisman M, Bambra C, Kunst AE (2008). Health inequalities according to educational level in different welfare regimes: a comparison of 23 European countries. Sociol Health Illn.

[24] European Foundation for the Improvement of Living and Working Conditions. Trends in quality of work in the Netherlands. 2007. http://eurofound.europa.eu/sites/default/files/ef_files/ewco/surveys/NL0601SR01/NL0601SR01.pdf. (Accessed 11 December 2014).

[25] Exworthy M, Berney L, Powell M (2002). ‘How great expectations in Westminster may be dashed locally’: the local implementation of national policy on health inequalities. Policy Polit.

[26] Giskes K, Kunst AE, Benach J, Borrell C, Costa G, Dahl E (2005). Trends in smoking behaviour between 1985 and 2000 in nine European countries by education. J Epidemiol Community Health.

[27] Horton R (2002). What the UK government is (not) doing about health inequalities. Lancet.

[28] House of Commons. Health inequalities. London: Health Committee of the House of Commons; 2009. http://www.publications.parliament.uk/pa/cm200809/cmselect/cmhealth/286/28602.htm. (Accessed 02 Feb 2015).

[29] Hu Y, van Lenthe FJ, Borsboom GJ, Looman CWN, Bopp M, Burström B, Dzúrová D, Ekholm O, Klumbiene J, Lahelma E, Leinsalu M, Regidor E, Santana P, de Gelder R, Mackenbach JP (2016). Trends in socioeconomic inequalities in self-assessed health in 17 European countries between 1990 and 2010. Journal of Epidemiology and Community Health.

[30] Hunter DJ, Popay J, Tannahill C, Whitehead M (2010). Getting to grips with health inequalities at last?. Br Med J.

[31] Italian National Institute of Statistics (ISTAT). Condizioni di salute e ricorso ai servizi sanitari [Health and Health Care Utilization]. Italian National Institute of Statistics, http://www.istat.it/it/archivio/5471. (Accessed 12 December 2014).

[32] Italian National Institute of Statistics (ISTAT). Multipurpose Family Survey. Italian National Institute of Statistics, http://www.istat.it/it/archivio/4394 (Accessed 16 July 2015).

[33] Italian National Institute of Statistics (ISTAT). Multipurpose Family survey: aspects of daily life. Italian National Institute of Statistics, http://www.istat.it/it/archivio/4630 (Accessed 16 July 2015).

[34] Judge K, Bauld L (2006). Learning from policy failure? Health action zones in England. Eur J Public Health.

[35] Judge K, Platt S, Costongs C, & Jurzcak K. Health inequalities: a challenge for Europe. An independent, expert report commissioned by, and published under the auspices of, UK Presidency of the EU. 2006. http://ec.europa.eu/health/ph_determinants/socio_economics/documents/ev_060302_rd05_en.pdf (Accessed 02 Feb 2015).

[36] Katikireddi SV, Niedzwiedz CL, & Popham F. Trends in population mental health before and after the 2008 recession: a repeat cross-sectional analysis of the 1991–2010 Health Surveys of England. BMJ Open. 2012;2:e001790. doi:10.1136/bmjopen-2012-001790. 10.1136/bmjopen-2012-001790 PMC3488736 23075569

[37] Kunst AE, Bos V, Lahelma E, Bartley M, Lissau I, Regidor E (2005). Trends in socioeconomic inequalities in self-assessed health in 10 European countries. Int J Epidemiol.

[38] Macintyre S, Chalmers I, Horton R, Smith R (2001). Using evidence to inform health policy: case studs. Br Med J.

[39] Mackenbach JP, Siegrist J, Marmot M (2006). Socio-economic inequalities in health in western Europe: from description to explanation to intervention. Social inequalities in health: New evidence and policy implications.

[40] Mackenbach JP (2010). Has the English strategy to reduce health inequalities failed?. Soc Sci Med.

[41] Mackenbach JP (2011). Can we reduce health inequalities? An analysis of the English strategy (1997–2010). J Epidemiol Community Health.

[42] Mackenbach JP (2011). The English strategy to reduce health inequalities. Lancet.

[43] Mackenbach JP, Gulliford M, Detels R, Karim QA, Tan CC (2015). Socioeconomic inequalities in health in high‐income countries: the facts and the options. Oxford Textbook of Global Public Health.

[44] Mackenbach JP, Bakker MJ (2003). European Network on I & Policies to Reduce Inequalities in H. Tackling socioeconomic inequalities in health: analysis of European experiences. Lancet.

[45] Mackenbach JP, Bos V, Andersen O, Cardano M, Costa G, Harding S (2003). Widening socioeconomic inequalities in mortality in six Western European countries. Int J Epidemiol.

[46] Mackenbach JP, Kulhanova I, Menvielle G, Bopp M, Borrell C, Costa G, et al. Trends in inequalities in premature mortality: a study of 3.2 million deaths in 13 European countries. J Epidemiol Community Health. 2014. doi:10.1136/jech-2014-204319. 10.1136/jech-2014-204319 24964740

[47] Mackenbach JP, Kunst AE, Cavelaars AE, Groenhof F, Geurts JJ (1997). Socioeconomic inequalities in morbidity and mortality in western Europe. The EU Working Group on Socioeconomic Inequalities in Health. Lancet.

[48] Mackenbach JP, Stirbu I, Roskam AJ, Schaap MM, Menvielle G, Leinsalu M (2008). Socioeconomic inequalities in health in 22 European countries. N Engl J Med.

[49] Mackenbach JP, Stronks K (2002). A strategy for tackling health inequalities in the Netherlands. BMJ.

[50] Mackenbach JP, Stronks K (2004). The development of a strategy for tackling health inequalities in the Netherlands. Int J Equity Health.

[51] Manderbacka K (1998). Examining what self-rated health question is understood to mean by respondents. Scand J Soc Med.

[52] Melhuish E, Belsky J, Leyland AH, Barnes J, National Evaluation Of Sure Start Research, T. (2008). Effects of fully-established Sure Start Local Programmes on 3-year-old children and their families living in England: a quasi-experimental observational study. Lancet.

[53] Ministry of Social Affairs and Health. National Action Plan to Reduce Health Inequalities 2008–2011. Helsinki. 2008. https://julkaisut.valtioneuvosto.fi/bitstream/handle/10024/71185/Julk200825.pdf?sequence=1. Accessed 18 Aug 2016.

[54] Lechner M (2011). The Estimation of Causal Effects by Difference-in-Difference Methods. Found Trends Econ.

[55] National Institute for Health and Welfare. Population studies: Survey on Health Behaviour and Health among the Finiish Adult Population (AVTK), Health Behaviour and Health among the Finnish Retirement-Age Populatin (EVTK). https://www.thl.fi/en/web/thlfi-en/research-and-expertwork/population-studies (Accessed 16 July 2015).

[56] Peters F, Nusselder WJ, Reibling N, Wegner-Siegmundt C, Mackenbach JP (2015). Quantifying the contribution of changes in healthcare expenditures and smoking to the reversal of the trend in life expectancy in the Netherlands. BMC Public Health.

[57] Suhrcke M, Stuckler D (2012). Will the recession be bad for our health? It depends. Soc Sci Med.

[58] Wanner P. Migration Trends in Europe. European Population Papers Series No. 7. European Population Committee, Council of Europe. 2002 http://www2.unine.ch/repository/default/content/sites/sfm/files/shared/pub/o/o_09.pdf (Accessed 18 August 2016).

[59] Whitehead M (1998). Diffusion of ideas on social inequalities in health: a European perspective. Milbank Q.

[60] Wooldridge JM. Introductory Econometrics: A Modern Approach: Cengage Learning; 5 edition. Mason, Michigan. 2012.

