# Supplementary material for: Did the English strategy reduce inequalities in health? A difference-in-difference analysis comparing England with three other European countries
Source: BMC Public Health. 2016 Aug 24;16(1):865. doi: 10.1186/s12889-016-3505-z (PMC4995654; doi:10.1186/s12889-016-3505-z)
Supplement: Additional file 1: — Web appendix. (DOCX 79 kb) [file 12889_2016_3505_MOESM1_ESM.docx]

**Web appendix**

**Details on the models, coding of the variables and the full results**

1. The two-way interaction model:

$${outcome}_{ist}=\beta_{0}+\beta_{1}{endyear}_{t}+\beta_{2}{policyperiod}_{s}+\beta_{3}{endyear}_{t}*{policyperiod}_{s}+{age}_{ist}+{sex}_{ist}$$

| Independent Variables | Definitions/Coding |
| --- | --- |
| *endyear* | For the period 1990-2000:  *endyear*=1 if year=2000 *endyear*=0 if year=1990;  For the period 2000-2010:  *endyear*=1 if year=2010 *endyear*=0 if year=2000^a^ |
| *policyperiod* | *policyperiod*=1 if the period is 2000-2010; *policyperiod*=0 if the period is 1990-2000^a^ |
| *endyear*policyperiod* | the interaction between *endyear* and *policyperiod* |
| *age* | continuous variable |
| *sex* | *sex*=1 for males; *sex*=2 for females |

1. Please note that since the period 1990-2000 was used as an independent control condition for the treatment condition 2000-2010, data from year 2000 were used twice (both as the starting year of the treatment period and as the ending year of the control period). In this way, we could give the data from year 2000 different values for both variables “*endyear*” and “*policyperiod*”, depending on whether the year 2000 was regarded as the ending year of the control period or as the starting year of the treatment period.

As for the specific year, year 2000 was always considered as the year when the English strategy was not generally implemented (no matter whether it was considered as the ending year of 1990-2000 or the starting year of 2000-2010). Being the ending year of 2000-2010 (the treatment period), year 2010 was the only year that was considered as the year when the English strategy potentially had effects.

The full results of the two-way interaction models conducted among low-educated people in England are:

|  | Odds ratios (logistic) | | | |  | Coefficients (linear) |
| --- | --- | --- | --- | --- | --- | --- |
| Variables | Less-than-good SAH | LONGHP | smoker | obesity |  | amount of smoking |
|  |  |  |  |  |  |  |
| policyperiod | 1.307*** | 1.325*** | 0.949 | 1.501*** |  | -0.453 |
|  | (0.0736) | (0.0738) | (0.0551) | (0.103) |  | (0.411) |
| endyear | 1.307*** | 1.325*** | 0.949 | 1.501*** |  | -0.453 |
|  | (0.0736) | (0.0738) | (0.0551) | (0.103) |  | (0.411) |
| policyperiod*endyear | 0.762*** | 0.780*** | 0.822** | 0.970 |  | -0.643 |
|  | (0.0639) | (0.0654) | (0.0732) | (0.0967) |  | (0.631) |
| age | 1.024*** | 1.037*** | 0.968*** | 1.013*** |  | -0.0387*** |
|  | (0.00131) | (0.00131) | (0.00123) | (0.00143) |  | (0.00906) |
| sex | 0.975 | 0.929* | 0.884*** | 1.363*** |  | -2.712*** |
|  | (0.0408) | (0.0387) | (0.0387) | (0.0690) |  | (0.318) |
| Constant | 0.141*** | 0.146*** | 3.546*** | 0.0720*** |  | 22.26*** |
|  | (0.0143) | (0.0145) | (0.361) | (0.00865) |  | (0.694) |
|  |  |  |  |  |  |  |
| Observations | 10,336 | 10,341 | 10,316 | 8,875 |  | 3,377 |
| Robust standard errors in parentheses, *** p<0.01, ** p<0.05, * p<0.1 | | | | | | |

We did not use the clustered standard errors in our analysis since the clustered standard errors can be biased if the number of clusters is small ([Angrist & Pischke, 2009](#_ENREF_1)), and the cure can be worse than the disease ([Nichols & Schaffer, 2007](#_ENREF_50)). There is no agreement on the required number of clusters for reliable inference. Angrist and Pischke semi-jokingly gave the number of 42 as the minimum number of clusters. In our study, there are only 2 countries at most in each regression, which is far from enough for the clustered standard errors to be reliable. Therefore, we only used the robust standard errors to account for potential heteroscedasticity.

However, given that clustered standard errors are generally larger than normal or robust standard errors, we believe that using clustered standard errors will not change our general conclusions since most of the four-way interactions were already insignificant.

2. The three-way interaction model:

$${outcome}_{ist}=\beta_{0}+\beta_{1}e{ndyear}_{t}+\beta_{2}{policyperiod}_{s}+\beta_{3}e{ndyear}_{t}*{policyperiod}_{s}+\beta_{4}{ledu}_{ist}+\beta_{5}{ledu}_{ist}*e{ndyear}_{t}+\beta_{6}{ledu}_{ist}*{policyperiod}_{s}+\beta_{7}{ledu}_{ist}*e{ndyear}_{t}*{policyperiod}_{s}+X_{ist}$$

The coding for *endyear, policyperiod, age* and *sex* is exactly the same as that in the two-way interaction model. The dummy variable *ledu* and its interactions were additionally added. Specifically, *ledu*=1 for low-educated people, and *ledu*=0 for high-educated people. Middle-educated group was excluded from this analysis.

The full results of the three-way interaction models conducted for England are:

|  | Odds ratios (logistic) | | | |  | Coefficients (linear) |
| --- | --- | --- | --- | --- | --- | --- |
| Variables | Less-than-good SAH | LONGHP | smoker | obesity |  | amount of smoking |
|  |  |  |  |  |  |  |
| policyperiod | 1.464*** | 1.112 | 0.955 | 1.657*** |  | 0.206 |
|  | (0.162) | (0.0871) | (0.0890) | (0.187) |  | (0.765) |
| endyear | 1.464*** | 1.112 | 0.955 | 1.657*** |  | 0.206 |
|  | (0.162) | (0.0871) | (0.0890) | (0.187) |  | (0.765) |
| ledu | 3.061*** | 1.209*** | 3.239*** | 1.535*** |  | 4.821*** |
|  | (0.308) | (0.0887) | (0.273) | (0.164) |  | (0.654) |
| policyperiod*endyear | 0.624*** | 0.818** | 0.694*** | 0.780* |  | -1.953* |
|  | (0.0862) | (0.0822) | (0.0865) | (0.108) |  | (1.009) |
| policyperiod*ledu | 0.891 | 1.191* | 0.988 | 0.899 |  | -0.692 |
|  | (0.111) | (0.115) | (0.108) | (0.119) |  | (0.869) |
| endyear*ledu | 0.891 | 1.191* | 0.988 | 0.899 |  | -0.692 |
|  | (0.111) | (0.115) | (0.108) | (0.119) |  | (0.869) |
| policyperiod*endyear*ledu | 1.220 | 0.952 | 1.189 | 1.250 |  | 1.299 |
|  | (0.197) | (0.125) | (0.182) | (0.213) |  | (1.191) |
| age | 1.025*** | 1.039*** | 0.969*** | 1.014*** |  | -0.00674 |
|  | (0.00115) | (0.00108) | (0.00109) | (0.00119) |  | (0.00822) |
| sex | 0.994 | 0.994 | 0.808*** | 1.175*** |  | -2.739*** |
|  | (0.0350) | (0.0315) | (0.0287) | (0.0459) |  | (0.267) |
| Constant | 0.0412*** | 0.0990*** | 1.185* | 0.0556*** |  | 15.98*** |
|  | (0.00471) | (0.00884) | (0.117) | (0.00674) |  | (0.766) |
|  |  |  |  |  |  |  |
| Observations | 18,245 | 18,250 | 18,218 | 15,878 |  | 4,711 |
| Robust standard errors in parentheses, *** p<0.01, ** p<0.05, * p<0.1 | | | | | | |

The full results of the three-way interaction models conducted for Finland are:

|  | Odds ratios (logistic) | | |  | Coefficients (linear) |
| --- | --- | --- | --- | --- | --- |
| Variables | Less-than-good SAH | smoker | obesity |  | amount of smoking |
|  |  |  |  |  |  |
| policyperiod | 0.978 | 0.935 | 1.882** |  | 0.177 |
|  | (0.151) | (0.141) | (0.503) |  | (1.332) |
| endyear | 0.978 | 0.935 | 1.882** |  | 0.177 |
|  | (0.151) | (0.141) | (0.503) |  | (1.332) |
| ledu | 2.588*** | 2.062*** | 2.554*** |  | 4.793*** |
|  | (0.352) | (0.273) | (0.635) |  | (1.188) |
| policyperiod*endyear | 1.133 | 0.919 | 0.586* |  | -2.217 |
|  | (0.220) | (0.182) | (0.183) |  | (1.621) |
| policyperiod*ledu | 0.868 | 0.793 | 0.588* |  | 0.0518 |
|  | (0.149) | (0.139) | (0.168) |  | (1.519) |
| endyear*ledu | 0.868 | 0.793 | 0.588* |  | 0.0518 |
|  | (0.149) | (0.139) | (0.168) |  | (1.519) |
| policyperiod*endyear*ledu | 0.777 | 1.275 | 1.896* |  | 1.228 |
|  | (0.173) | (0.308) | (0.652) |  | (2.003) |
| age | 1.038*** | 0.969*** | 1.021*** |  | 0.0640*** |
|  | (0.00169) | (0.00168) | (0.00208) |  | (0.0180) |
| sex | 0.997 | 0.555*** | 1.306*** |  | -3.934*** |
|  | (0.0460) | (0.0305) | (0.0807) |  | (0.434) |
| Constant | 0.0595*** | 2.420*** | 0.0143*** |  | 14.03*** |
|  | (0.00931) | (0.378) | (0.00379) |  | (1.441) |
|  |  |  |  |  |  |
| Observations | 8,864 | 8,548 | 8,762 |  | 1,525 |
| Robust standard errors in parentheses, *** p<0.01, ** p<0.05, * p<0.1 | | | | | |

The full results of the three-way interaction models conducted for the Netherlands are:

|  | Odds ratios (logistic) | | |  | Coefficients (linear) |
| --- | --- | --- | --- | --- | --- |
| Variables | Less-than-good SAH | LONGHP | smoker |  | amount of smoking |
|  |  |  |  |  |  |
| policyperiod | 0.841 | 1.410*** | 0.640*** |  | -3.583*** |
|  | (0.106) | (0.149) | (0.0672) |  | (1.012) |
| endyear | 0.841 | 1.410*** | 0.640*** |  | -3.583*** |
|  | (0.106) | (0.149) | (0.0672) |  | (1.012) |
| ledu | 1.938*** | 1.483*** | 1.663*** |  | 1.465* |
|  | (0.215) | (0.149) | (0.156) |  | (0.791) |
| policyperiod*endyear | 0.836 | 0.716** | 1.195 |  | 2.836** |
|  | (0.139) | (0.0947) | (0.167) |  | (1.367) |
| policyperiod*ledu | 1.174 | 0.973 | 1.164 |  | 0.567 |
|  | (0.164) | (0.118) | (0.144) |  | (1.157) |
| endyear*ledu | 1.174 | 0.973 | 1.164 |  | 0.567 |
|  | (0.164) | (0.118) | (0.144) |  | (1.157) |
| policyperiod*endyear*ledu | 1.177 | 1.163 | 0.995 |  | 0.816 |
|  | (0.221) | (0.181) | (0.165) |  | (1.589) |
| age | 1.033*** | 1.031*** | 0.983*** |  | 0.00593 |
|  | (0.00115) | (0.00104) | (0.00111) |  | (0.0109) |
| sex | 1.211*** | 1.235*** | 0.672*** |  | -1.059*** |
|  | (0.0467) | (0.0423) | (0.0257) |  | (0.369) |
| Constant | 0.0335*** | 0.0556*** | 1.968*** |  | 16.62*** |
|  | (0.00416) | (0.00617) | (0.212) |  | (0.981) |
|  |  |  |  |  |  |
| Observations | 16,496 | 16,492 | 13,438 |  | 3,092 |
| Robust standard errors in parentheses, *** p<0.01, ** p<0.05, * p<0.1 | | | | | |

The full results of the three-way interaction models conducted for Italy are:

|  | Odds ratios (logistic) | |  | Coefficients (linear) |
| --- | --- | --- | --- | --- |
| Variables | smoker | obesity |  | amount of smoking |
|  |  |  |  |  |
| policyperiod | 0.783*** | 1.405*** |  | -1.025*** |
|  | (0.0409) | (0.184) |  | (0.369) |
| endyear | 0.783*** | 1.405*** |  | -1.025*** |
|  | (0.0409) | (0.184) |  | (0.369) |
| ledu | 0.962 | 2.641*** |  | 1.873*** |
|  | (0.0438) | (0.315) |  | (0.328) |
| policyperiod*endyear | 1.103 | 1.063 |  | -1.025** |
|  | (0.0779) | (0.165) |  | (0.484) |
| policyperiod*ledu | 1.190*** | 0.955 |  | -0.408 |
|  | (0.0648) | (0.127) |  | (0.394) |
| endyear*ledu | 1.190*** | 0.955 |  | -0.408 |
|  | (0.0648) | (0.127) |  | (0.394) |
| policyperiod*endyear*ledu | 0.967 | 0.761* |  | 0.772 |
|  | (0.0724) | (0.121) |  | (0.522) |
| age | 0.980*** | 1.026*** |  | 0.00574** |
|  | (0.000271) | (0.000388) |  | (0.00285) |
| sex | 0.397*** | 0.888*** |  | -4.437*** |
|  | (0.00416) | (0.0126) |  | (0.0830) |
| Constant | 3.758*** | 0.0115*** |  | 20.73*** |
|  | (0.177) | (0.00139) |  | (0.346) |
|  |  |  |  |  |
| Observations | 218,223 | 213,977 |  | 53,436 |
| Robust standard errors in parentheses, *** p<0.01, ** p<0.05, * p<0.1 | | | | |

3. The four-way interaction model:

$${outcome}_{istj}=the three-way interaction model*(1+england dummy)=\left( \beta_{0}+\beta_{1}{endyear}_{tj}+\beta_{2}{policyperiod}_{sj}+\beta_{3}{endyear}_{tj}*{policyperiod}_{sj}+\beta_{4}{ledu}_{istj}+\beta_{5}{ledu}_{istj}*{endyear}_{tj}+\beta_{6}{ledu}_{istj}*{policyperiod}_{sj}+\beta_{7}{ledu}_{istj}*{endyear}_{tj}*{policyperiod}_{sj}+X_{istj} \right)+(\beta_{0}^{'}+\beta_{1}^{'}{endyear}_{tj}+\beta_{2}^{'}{policyperiod}_{sj}+\beta_{3}^{'}{endyear}_{tj}*{policyperiod}_{sj}+\beta_{4}^{'}{ledu}_{istj}+\beta_{5}^{'}{ledu}_{istj}*{endyear}_{tj}+\beta_{6}^{'}{ledu}_{istj}*{policyperiod}_{sj}+\beta_{7}^{'}{ledu}_{istj}*{endyear}_{tj}*{policyperiod}_{sj}+X_{istj})*{england}_{j}$$

The coding for *endyear*, *policyperiod*, *ledu*, *age* and *sex* is exactly the same as that in the two-way and three-way interaction models. But since we included data from both England and one of the comparison countries in the regression, we additionally added a dummy for England and its interaction terms into the regression. Specifically, *england=*1 for data from England, and *england*=0 for data from the comparison countries.

The full results of the four-way interaction models comparing the changes in trends in health inequalities between 2000-2010 and 1990-2000 in England and those in Finland are:

|  | Odds ratios (logistic) | | |  | Coefficients (linear) |
| --- | --- | --- | --- | --- | --- |
| Variables | Less-than-good SAH | smoker | obesity |  | amount of smoking |
|  |  |  |  |  |  |
| policyperiod | 0.978 | 0.935 | 1.882** |  | 0.177 |
|  | (0.151) | (0.141) | (0.503) |  | (1.330) |
| endyear | 0.978 | 0.935 | 1.882** |  | 0.177 |
|  | (0.151) | (0.141) | (0.503) |  | (1.330) |
| ledu | 2.588*** | 2.062*** | 2.554*** |  | 4.793*** |
|  | (0.352) | (0.273) | (0.635) |  | (1.186) |
| policyperiod*endyear | 1.133 | 0.919 | 0.586* |  | -2.217 |
|  | (0.220) | (0.182) | (0.183) |  | (1.618) |
| policyperiod*ledu | 0.868 | 0.793 | 0.588* |  | 0.0518 |
|  | (0.149) | (0.139) | (0.168) |  | (1.517) |
| endyear*ledu | 0.868 | 0.793 | 0.588* |  | 0.0518 |
|  | (0.149) | (0.139) | (0.168) |  | (1.517) |
| policyperiod*endyear*ledu | 0.777 | 1.275 | 1.896* |  | 1.228 |
|  | (0.173) | (0.308) | (0.652) |  | (1.999) |
| england | 0.693* | 0.490*** | 3.890*** |  | 1.948 |
|  | (0.134) | (0.0906) | (1.133) |  | (1.630) |
| england*policyperiod | 1.498** | 1.021 | 0.881 |  | 0.0294 |
|  | (0.285) | (0.181) | (0.255) |  | (1.534) |
| england*endyear | 1.498** | 1.021 | 0.881 |  | 0.0294 |
|  | (0.285) | (0.181) | (0.255) |  | (1.534) |
| england*ledu | 1.182 | 1.571*** | 0.601* |  | 0.0282 |
|  | (0.200) | (0.246) | (0.163) |  | (1.355) |
| england*policyperiod*endyear | 0.551** | 0.755 | 1.330 |  | 0.263 |
|  | (0.131) | (0.177) | (0.455) |  | (1.908) |
| england*policyperiod*ledu | 1.026 | 1.247 | 1.530 |  | -0.744 |
|  | (0.217) | (0.258) | (0.482) |  | (1.748) |
| england*endyear*ledu | 1.026 | 1.247 | 1.530 |  | -0.744 |
|  | (0.217) | (0.258) | (0.482) |  | (1.748) |
| england*policyperiod*endyear*ledu | 1.571 | 0.933 | 0.659 |  | 0.0712 |
|  | (0.433) | (0.267) | (0.253) |  | (2.328) |
| age | 1.038*** | 0.969*** | 1.021*** |  | 0.0640*** |
|  | (0.00169) | (0.00168) | (0.00208) |  | (0.0180) |
| sex | 0.997 | 0.555*** | 1.306*** |  | -3.934*** |
|  | (0.0460) | (0.0305) | (0.0807) |  | (0.434) |
| england*age | 0.988*** | 1.000 | 0.993*** |  | -0.0707*** |
|  | (0.00195) | (0.00206) | (0.00233) |  | (0.0198) |
| england*sex | 0.997 | 1.456*** | 0.899 |  | 1.195** |
|  | (0.0579) | (0.0952) | (0.0657) |  | (0.509) |
| Constant | 0.0595*** | 2.420*** | 0.0143*** |  | 14.03*** |
|  | (0.00931) | (0.378) | (0.00379) |  | (1.438) |
| Observations | 27,109 | 26,766 | 24,640 |  | 6,236 |
| Robust standard errors in parentheses, *** p<0.01, ** p<0.05, * p<0.1 | | | | | |

The full results of the four-way interaction models comparing the changes in trends in health inequalities between 2000-2010 and 1990-2000 in England and those in the Netherlands are:

|  | Odds ratios (logistic) | | |  | Coefficients (linear) |
| --- | --- | --- | --- | --- | --- |
| Variables | Less-than-good SAH | LONGHP | smoker |  | amount of smoking |
|  |  |  |  |  |  |
| policyperiod | 0.841 | 1.410*** | 0.640*** |  | -3.583*** |
|  | (0.106) | (0.149) | (0.0672) |  | (1.012) |
| endyear | 0.841 | 1.410*** | 0.640*** |  | -3.583*** |
|  | (0.106) | (0.149) | (0.0672) |  | (1.012) |
| ledu | 1.938*** | 1.483*** | 1.663*** |  | 1.465* |
|  | (0.215) | (0.149) | (0.156) |  | (0.790) |
| policyperiod*endyear | 0.836 | 0.716** | 1.195 |  | 2.836** |
|  | (0.139) | (0.0947) | (0.167) |  | (1.366) |
| policyperiod*ledu | 1.174 | 0.973 | 1.164 |  | 0.567 |
|  | (0.164) | (0.118) | (0.144) |  | (1.156) |
| endyear*ledu | 1.174 | 0.973 | 1.164 |  | 0.567 |
|  | (0.164) | (0.118) | (0.144) |  | (1.156) |
| policyperiod*endyear*ledu | 1.177 | 1.163 | 0.995 |  | 0.816 |
|  | (0.221) | (0.181) | (0.165) |  | (1.589) |
| england | 1.232 | 1.779*** | 0.602*** |  | -0.639 |
|  | (0.208) | (0.253) | (0.0879) |  | (1.244) |
| england*policyperiod | 1.740*** | 0.789* | 1.492*** |  | 3.789*** |
|  | (0.291) | (0.104) | (0.209) |  | (1.268) |
| england*endyear | 1.740*** | 0.789* | 1.492*** |  | 3.789*** |
|  | (0.291) | (0.104) | (0.209) |  | (1.268) |
| england*ledu | 1.580*** | 0.816 | 1.948*** |  | 3.356*** |
|  | (0.237) | (0.102) | (0.246) |  | (1.026) |
| england*policyperiod*endyear | 0.746 | 1.142 | 0.581*** |  | -4.789*** |
|  | (0.161) | (0.190) | (0.109) |  | (1.699) |
| england*policyperiod*ledu | 0.758 | 1.224 | 0.849 |  | -1.260 |
|  | (0.142) | (0.190) | (0.140) |  | (1.447) |
| england*endyear*ledu | 0.758 | 1.224 | 0.849 |  | -1.260 |
|  | (0.142) | (0.190) | (0.140) |  | (1.447) |
| england*policyperiod*endyear*ledu | 1.037 | 0.819 | 1.195 |  | 0.483 |
|  | (0.257) | (0.167) | (0.270) |  | (1.986) |
| age | 1.033*** | 1.031*** | 0.983*** |  | 0.00593 |
|  | (0.00115) | (0.00104) | (0.00111) |  | (0.0109) |
| sex | 1.211*** | 1.235*** | 0.672*** |  | -1.059*** |
|  | (0.0467) | (0.0423) | (0.0257) |  | (0.369) |
| england*age | 0.992*** | 1.007*** | 0.986*** |  | -0.0127 |
|  | (0.00157) | (0.00146) | (0.00157) |  | (0.0136) |
| england*sex | 0.821*** | 0.805*** | 1.202*** |  | -1.680*** |
|  | (0.0429) | (0.0375) | (0.0627) |  | (0.455) |
| Constant | 0.0335*** | 0.0556*** | 1.968*** |  | 16.62*** |
|  | (0.00415) | (0.00617) | (0.212) |  | (0.981) |
| Observations | 34,741 | 34,742 | 31,656 |  | 7,803 |
| Robust standard errors in parentheses, *** p<0.01, ** p<0.05, * p<0.1 | | | | | |

The full results of the four-way interaction models comparing the changes in trends in health inequalities between 2000-2010 and 1990-2000 in England and those in Italy are:

|  | Odds ratios (logistic) | |  | Coefficients (linear) |
| --- | --- | --- | --- | --- |
| Variables | smoker | obesity |  | amount of smoking |
|  |  |  |  |  |
| policyperiod | 0.783*** | 1.405*** |  | -1.025*** |
|  | (0.0409) | (0.184) |  | (0.369) |
| endyear | 0.783*** | 1.405*** |  | -1.025*** |
|  | (0.0409) | (0.184) |  | (0.369) |
| ledu | 0.962 | 2.641*** |  | 1.873*** |
|  | (0.0438) | (0.315) |  | (0.328) |
| policyperiod*endyear | 1.103 | 1.063 |  | -1.025** |
|  | (0.0779) | (0.165) |  | (0.484) |
| policyperiod*ledu | 1.190*** | 0.955 |  | -0.408 |
|  | (0.0648) | (0.127) |  | (0.394) |
| endyear*ledu | 1.190*** | 0.955 |  | -0.408 |
|  | (0.0648) | (0.127) |  | (0.394) |
| policyperiod*endyear*ledu | 0.967 | 0.761* |  | 0.772 |
|  | (0.0724) | (0.121) |  | (0.522) |
| england | 0.315*** | 4.816*** |  | -4.755*** |
|  | (0.0345) | (0.823) |  | (0.840) |
| england*policyperiod | 1.220* | 1.180 |  | 1.231 |
|  | (0.130) | (0.204) |  | (0.849) |
| england*endyear | 1.220* | 1.180 |  | 1.231 |
|  | (0.130) | (0.204) |  | (0.849) |
| england*ledu | 3.367*** | 0.581*** |  | 2.948*** |
|  | (0.323) | (0.0930) |  | (0.731) |
| england*policyperiod*endyear | 0.629*** | 0.734 |  | -0.929 |
|  | (0.0902) | (0.153) |  | (1.118) |
| england*policyperiod*ledu | 0.830 | 0.942 |  | -0.285 |
|  | (0.102) | (0.177) |  | (0.953) |
| england*endyear*ledu | 0.830 | 0.942 |  | -0.285 |
|  | (0.102) | (0.177) |  | (0.953) |
| england*policyperiod*endyear*ledu | 1.230 | 1.642** |  | 0.527 |
|  | (0.209) | (0.383) |  | (1.299) |
| age | 0.980*** | 1.026*** |  | 0.00574** |
|  | (0.000271) | (0.000388) |  | (0.00285) |
| sex | 0.397*** | 0.888*** |  | -4.437*** |
|  | (0.00416) | (0.0126) |  | (0.0830) |
| england*age | 0.990*** | 0.988*** |  | -0.0125 |
|  | (0.00114) | (0.00121) |  | (0.00869) |
| england*sex | 2.036*** | 1.323*** |  | 1.699*** |
|  | (0.0754) | (0.0550) |  | (0.280) |
| Constant | 3.758*** | 0.0115*** |  | 20.73*** |
|  | (0.177) | (0.00139) |  | (0.346) |
| Observations | 236,441 | 229,855 |  | 58,147 |
| Robust standard errors in parentheses, *** p<0.01, ** p<0.05, * p<0.1 | | | | |

Angrist, J. D., & Pischke, J. S. (2009). Mostly harmless econometrics: Princeton University Press.

Nichols, A., & Schaffer, M. (2007). Clustered errors in Stata. United Kingdom Stata Users' Group Meetings, http://repec.org/usug2007/crse.pdf (accessed 7th July 2015).

**Table A1 Information on data collection and health outcome measures**

| Country | Survey year | Sampling strategy | Response rate | Question and answer for SAH | Question and answer for LONGHP | Measurement of smoking status | Measurement of amount of smoking | The way of collecting height /weight |
| --- | --- | --- | --- | --- | --- | --- | --- | --- |
| England | 1991-1992 | multi-stage stratified random sample | 84% | How is your health in general?  1= fair/bad/very bad  0= very good/good | Do you have any longstanding illness, disability or infirmity?  1=Yes 0=No | 1= current smoker  0=ex- or non-smoker | Number of cigarettes per day | Measured |
|  | 2000 | multi-stage stratified random sample | 75% | How is your health in general?  1= fair/bad/very bad  0= very good/good | Do you have any longstanding illness, disability or infirmity?  1=Yes 0=No | 1= current smoker  0=ex- or non-smoker | Number of cigarettes per day | Measured |
|  | 2010 | multi-stage stratified random sample | 66% | How is your health in general?  1= fair/bad/very bad  0= very good/good | Do you have any longstanding illness, disability or infirmity?  1=Yes 0=No | 1= current smoker  0=ex- or non-smoker | Number of cigarettes per day | Measured |
| Finland | 1989 | (stratified) random sample^a^ | 77%/87%^b^ | Assessment of own health status:  1= average/rather poor/poor  0= good/reasonably good | - | 1= current smoker  0=ex- or non-smoker | Number of cigarettes per day | Self-reported |
|  | 1999 | (stratified) random sample^a^ | 69%/75%^b^ | Assessment of own health status:  1= average/rather poor/poor  0= good/reasonably good | - | 1= current smoker  0=ex- or non-smoker | Number of cigarettes per day | Self-reported |
|  | 2009 | (stratified) random sample^a^ | 59%/73%^b^ | Assessment of own health status:  1= average/rather poor/poor  0= good/reasonably good | - | 1= current smoker  0=ex- or non-smoker | Number of cigarettes per day | Self-reported |
| The Netherlands | 1990 | three-stage sample: municipalities, addresses, people | 47% | In general, how do you experience your health condition?  1= fair/poor/very poor  0= very good/good | Do you suffer from any long-standing illness, infirmity, or disability?  1=Yes 0=No | 1= current smoker  0=ex- or non-smoker | Number of cigarettes per day | - |
|  | 2000 | two-stage sample: municipalities, people | 59% | How in general is your health condition?  1= fair/sometimes good sometimes poor/very poor  0= very good/good | Do you suffer from any long-standing illness, infirmity, or disability?  1=Yes 0=No | 1= current smoker  0=ex- or non-smoker | Number of cigarettes per day | - |
|  | 2009 | two-stage sample: municipalities, people | 63% | How in general is your health condition?  1= fair/sometimes good sometimes poor/very poor  0= very good/good | Do you suffer from any long-standing illness, infirmity, or disability?  1=Yes 0=No | 1= current smoker  0=ex- or non-smoker | Number of cigarettes per day | - |
| Italy | 1990 | two stage stratified sample | 96% | - | - | 1= current smoker  0=ex- or non-smoker | Number of cigarettes per day | Self-reported |
|  | 2000 | two stage stratified sample | 87% | - | - | 1= current smoker  0=ex- or non-smoker | Number of cigarettes per day | Self-reported |
|  | 2010 | two stage stratified sample | 82% | - | - | 1= current smoker  0=ex- or non-smoker | Number of cigarettes per day | Self-reported |

Notes:

a. The Finnish data used in this study are the data combined from the two Finish studies: “Health behaviour and health among Finnish adult population (AVTK)”, which includes respondents who are 15-64 years old, and “Health behaviour and health among the Finnish elderly (EVTK)”, which includes respondents who are older than 64 years. AVTK runs on a random sample of Finnish people aged 15-64 derived from the Population Register each year. EVTK runs on a random sample stratified by age and sex of Finnish people aged 65 or older every two years.

b. We report the response rates for AVTK/EVTK separately.

**Table A2 Classification of education in England**

| ISCED category | Education classification in the survey data | Percentage |
| --- | --- | --- |
|  |  |  |
| Year 1991-1992 | | |
| ISCED 0-2 (low-educated) | GCSE d-g or equiv; ungraded CSE; no qualification | 48% |
| ISCED 3-4 (middle-educated) | a levels or equiv; GCSE a-c or equiv | 34% |
| ISCED 5-6 (high-educated) | degree; prof not degree | 18% |
|  |  |  |
| Year 2000 | | |
| ISCED 0-2 (low-educated) | NVQ1/CSE other grade equiv; No qualification | 35% |
| ISCED 3-4 (middle-educated) | NVQ3/GCE A Level equiv; NVQ2/GCE O Level equiv | 37% |
| ISCED 5-6 (high-educated) | NVQ4/NVQ5/Degree or equiv; Higher ed below degree | 28% |
|  |  |  |
| Year 2010 | | |
| ISCED 0-2 (low-educated) | NVQ1/CSE other grade equiv; No qualification | 26% |
| ISCED 3-4 (middle-educated) | NVQ3/GCE A Level equiv; NVQ2/GCE O Level equiv | 40% |
| ISCED 5-6 (high-educated) | NVQ4/NVQ5/Degree or equiv; Higher ed below degree | 34% |

Table A3 Two-way interaction estimators comparing the trends in health between 1990s and 2000s, three-way interaction estimators comparing the trends in health inequalities between 1990s and 2000s, and four-way interaction estimators, comparing the three-way interaction estimators between countries, weighted results^a^

|  | Odds ratios (logistic) | | | | |  | Coefficients (linear) | |  |
| --- | --- | --- | --- | --- | --- | --- | --- | --- | --- |
|  | Less-than-good SAH | LONGHP | smoker | obesity |  | | | amount of smoking | |
| **1. two-way interaction estimators^a^** | | |  |  |  | | |  | |
| England (low-edu) | 0.75*** | 0.75*** | 0.80** | 0.95 |  | | | -0.91 | |
|  | (0.064) | (0.064) | (0.073) | (0.096) |  | | | (0.644) | |
| **2. three-way interaction estimators^b^** | |  |  |  |  | | |  | |
| England | 1.18 | 0.93 | 0.99 | 1.22 |  | | | 1.38 | |
|  | (0.195) | (0.124) | (0.108) | (0.210) |  | | | (1.199) | |
| Finland | 0.78 | - | 1.27 | 1.90* |  | | | 1.23 | |
|  | (0.173) | - | (0.308) | (0.652) |  | | | (2.003) | |
| the Netherlands | 1.17 | 1.02 | 1.00 | - |  | | | 1.45 | |
|  | (0.238) | (0.177) | (0.179) | - |  | | | (1.785) | |
| Italy | - | - | 0.93 | 0.81 |  | | | 0.39 | |
|  | - | - | (0.083) | (0.152) |  | | | (0.612) | |
|  |  |  |  |  |  | | |  | |
| **3. four-way interaction estimator^c^** | | | | | | | | | |
| England vs Finland | 1.52 | - | 0.89 | 0.64 |  | | | 0.15 | |
|  | (0.421) | - | (0.256) | (0.247) |  | | | (2.332) | |
| England vs the Netherlands | 1.01 | 0.90 | 1.14 | - |  | | | -0.08 | |
|  | (0.263) | (0.198) | (0.271) | - |  | | | (2.150) | |
| England vs Italy | - | - | 1.21 | 1.50 |  | | | 0.99 | |
|  | - | - | (0.219) | (0.381) |  | | | (1.345) | |
|  |  |  |  |  |  | | |  | |

a. Weights are available for England 2010, the Netherlands 1990/2000/2010 and Italy 1990/2000. Weights included in the Dutch survey 2000 and 2010 are expansion weights, which are scaled (i.e. divide each year by the mean weight) in order to be comparable to the other years and other countries.

b. Based on the two-way interaction analysis for low-educated people in England. An odds ratio below 1.00 or a negative linear coefficient indicates a larger health improvement in the period 2000-2010 than in the period 1990-2000.

c. Based on the three-way interaction analysis within each country. An odds ratio below 1.00 or a negative linear coefficient indicates a more favourable trend in health inequalities in the period 2000-2010 than in the period 1990-2000.

d. Based on the four-way interaction analysis for England and each of the comparison countries. An odds ratio below 1.00 or a negative linear coefficient indicates a more favourable change (between 1990-2000 and 2000-2010) in the trend in health inequalities in England as compared to the other country.

Robust standard errors in parentheses. *** p<0.01, ** p<0.05, * p<0.1

Table A4 Two-way interaction estimators comparing trends in health among low-educated people between 1990s and 2000s, within the three comparison countries

|  | Odds ratios (logistic) | | | |  | Coefficients (linear) |
| --- | --- | --- | --- | --- | --- | --- |
|  | Less-than-good SAH | LONGHP | smoker | obesity |  | amount of smoking |
| **two-way interaction estimators** | |  |  |  |  |  |
| Finland | 0.91 | - | 1.15 | 1.07 |  | -1.09 |
|  | (0.103) | - | (0.162) | (0.155) |  | (1.188) |
| the Netherlands | 0.99 | 0.84** | 1.20** | - |  | 3.82*** |
|  | (0.085) | (0.069) | (0.109) | - |  | (0.809) |
| Italy | - | - | 1.07** | 0.81*** |  | -0.25 |
|  | - | - | (0.027) | (0.028) |  | (0.194) |
| Robust standard errors in parentheses | | |  |  |  |  |
| *** p<0.01, ** p<0.05, * p<0.1 | |  |  |  |  |  |

Table A5 Two-way interaction estimators comparing the trends in mortality between the period late 1990s – late 2000s and the period late 1980s – late 1990s, three-way interaction estimators comparing the trends in mortality inequalities between the two periods, and four-way interaction estimators, comparing the three-way interaction estimators between England and Finland^a^

|  | Incidence-rate ratios (Poisson regression) |
| --- | --- |
|  | all-cause mortality |
| **1. two-way interaction estimators^a^** | |
| England (low- and mid- edu) | 0.91*** |
|  | (0.022) |
| **2. three-way interaction estimators^b^** | |
| England | 0.86** |
|  | (0.060) |
| Finland | 0.94 |
|  | (0.060) |
| **3. four-way interaction estimators^c^** | |
| England vs Finland | 0.91 |
|  | (0.086) |
|  |  |

a. We collected and harmonized mortality data by education among people aged 35-79 years in England and Finland between 1980s and 2000s. The period late 1980s – late 1990s was regarded as the period during which the English strategy had not yet been generally implemented and the period late 1990s – late 2000s was regarded as the period during which the main effects of the strategy could be expected. Due to data limitation, we could only classify two levels of education for England (“low+middle” vs “high”), thus the low and middle educated groups were also grouped together for Finland.

b. Based on a two-way interaction analysis for “low+middle” educated people in England. An incidence rate ratio below 1.00 indicates a larger reduction in all-cause mortality in the period late 1990s – late 2000s than in the period late 1980s – late 1990s.

c. Based on a three-way interaction analysis within each country. An incidence rate ratio below 1.00 indicates a more favourable trend in inequalities in mortality in the period late 1990s – late 2000s than in the period late 1980s – late 1990s.

d. Based on a four-way interaction analysis for England and Finland. An incidence rate ratio below 1.00 indicates a more favourable change (between late 1990s – late 2000s and late 1980s – late 1990s) in the trend in inequalities in mortality in England as compared to Finland.

Robust standard errors in parentheses. *** p<0.01, ** p<0.05, * p<0.1

Table A6 Two-way interaction estimators comparing the trends in health between 1990s and 2000s, three-way interaction estimators comparing the trends in health inequalities between 1990s and 2000s, and four-way interaction estimators, comparing the three-way interaction estimators between countries, among men

|  | Odds ratios (logistic) | | | | |  | Coefficients (linear) | |  |
| --- | --- | --- | --- | --- | --- | --- | --- | --- | --- |
|  | SAH | LONGHP | smoker | obesity |  | | | amount of smoking | |
| **1. two-way interaction estimators^a^** | |  |  |  |  | | |  | |
| England (low-edu) | 0.68*** | 0.70*** | 0.90 | 0.91 |  | | | -1.14 | |
|  | (0.086) | (0.088) | (0.119) | (0.142) |  | | | (1.052) | |
| **2. three-way interaction estimators^b^** | |  |  |  |  | | |  | |
| England | 1.32 | 0.79 | 1.25 | 1.34 |  | | | 2.36 | |
|  | (0.304) | (0.149) | (0.261) | (0.325) |  | | | (1.741) | |
| Finland | 0.86 | - | 1.55 | 1.85 |  | | | 4.13 | |
|  | (0.288) | - | (0.513) | (0.968) |  | | | (3.060) | |
| the Netherlands | 1.43 | 1.36 | 1.47* | - |  | | | 0.74 | |
|  | (0.392) | (0.310) | (0.335) | - |  | | | (2.506) | |
| Italy | - | - | 0.99 | 0.83 |  | | | -0.03 | |
|  | - | - | (0.097) | (0.161) |  | | | (0.754) | |
|  |  |  |  |  |  | | |  | |
| **3. four-way interaction estimator^c^** | | | | | | | | | |
| England vs Finland | 1.54 | - | 0.80 | 0.72 |  | | | -1.77 | |
|  | (0.627) | - | (0.314) | (0.415) |  | | | (3.514) | |
| England vs the Netherlands | 0.92 | 0.59* | 0.85 | - |  | | | 1.62 | |
|  | (0.329) | (0.173) | (0.263) | - |  | | | (3.050) | |
| England vs Italy | - | - | 1.26 | 1.61 |  | | | 2.39 | |
|  | - | - | (0.291) | (0.499) |  | | | (1.894) | |
|  |  |  |  |  |  | | |  | |

a. Based on the two-way interaction analysis for low-educated people in England. An odds ratio below 1.00 or a negative linear coefficient indicates a larger health improvement in the period 2000-2010 than in the period 1990-2000.

b. Based on the three-way interaction analysis within each country. An odds ratio below 1.00 or a negative linear coefficient indicates a more favourable trend in health inequalities in the period 2000-2010 than in the period 1990-2000.

c. Based on the four-way interaction analysis for England and each of the comparison countries. An odds ratio below 1.00 or a negative linear coefficient indicates a more favourable change (between 1990-2000 and 2000-2010) in the trend in health inequalities in England as compared to the other country.

Robust standard errors in parentheses. *** p<0.01, ** p<0.05, * p<0.1

Table A7 Two-way interaction estimators comparing the trends in health between 1990s and 2000s, three-way interaction estimators comparing the trends in health inequalities between 1990s and 2000s, and four-way interaction estimators, comparing the three-way interaction estimators between countries, among women

|  | Odds ratios (logistic) | | | | |  | Coefficients (linear) | |  |
| --- | --- | --- | --- | --- | --- | --- | --- | --- | --- |
|  | SAH | LONGHP | smoker | obesity |  | | | amount of smoking | |
| **1. two-way interaction estimators^a^** | |  |  |  |  | | |  | |
| England (low-edu) | 0.83 | 0.86 | 0.76** | 1.01 |  | | | -0.36 | |
|  | (0.093) | (0.096) | (0.092) | (0.131) |  | | | (0.743) | |
| **2. three-way interaction estimators^b^** | |  |  |  |  | | |  | |
| England | 1.08 | 1.13 | 1.13 | 1.07 |  | | | -0.73 | |
|  | (0.246) | (0.209) | (0.259) | (0.260) |  | | | (1.594) | |
| Finland | 0.70 | - | 1.18 | 1.87 |  | | | -2.38 | |
|  | (0.209) | - | (0.431) | (0.860) |  | | | (2.532) | |
| the Netherlands | 1.03 | 1.12 | 0.72 | - |  | | | 2.00 | |
|  | (0.270) | (0.241) | (0.180) | - |  | | | (2.021) | |
| Italy | - | - | 0.92 | 0.72 |  | | | 2.38*** | |
|  | - | - | (0.101) | (0.210) |  | | | (0.708) | |
|  |  |  |  |  |  | | |  | |
| **3. four-way interaction estimator^c^** | | | | | | | | | |
| England vs Finland | 1.54 | - | 0.96 | 0.57 |  | | | 1.66 | |
|  | (0.582) | - | (0.414) | (0.296) |  | | | (2.985) | |
| England vs the Netherlands | 1.04 | 1.01 | 1.58 | - |  | | | -2.73 | |
|  | (0.362) | (0.288) | (0.534) | - |  | | | (2.574) | |
| England vs Italy | - | - | 1.23 | 1.49 |  | | | -3.10* | |
|  | - | - | (0.313) | (0.566) |  | | | (1.742) | |
|  |  |  |  |  |  | | |  | |

a. Based on the two-way interaction analysis for low-educated people in England. An odds ratio below 1.00 or a negative linear coefficient indicates a larger health improvement in the period 2000-2010 than in the period 1990-2000.

b. Based on the three-way interaction analysis within each country. An odds ratio below 1.00 or a negative linear coefficient indicates a more favourable trend in health inequalities in the period 2000-2010 than in the period 1990-2000.

c. Based on the four-way interaction analysis for England and each of the comparison countries. An odds ratio below 1.00 or a negative linear coefficient indicates a more favourable change (between 1990-2000 and 2000-2010) in the trend in health inequalities in England as compared to the other country.

Robust standard errors in parentheses. *** p<0.01, ** p<0.05, * p<0.1
