# Supplementary figures and images for: Did the English strategy reduce inequalities in health? A difference-in-difference analysis comparing England with three other European countries
Source: BMC Public Health. 2016 Aug 24;16(1):865. doi: 10.1186/s12889-016-3505-z (PMC4995654; doi:10.1186/s12889-016-3505-z)

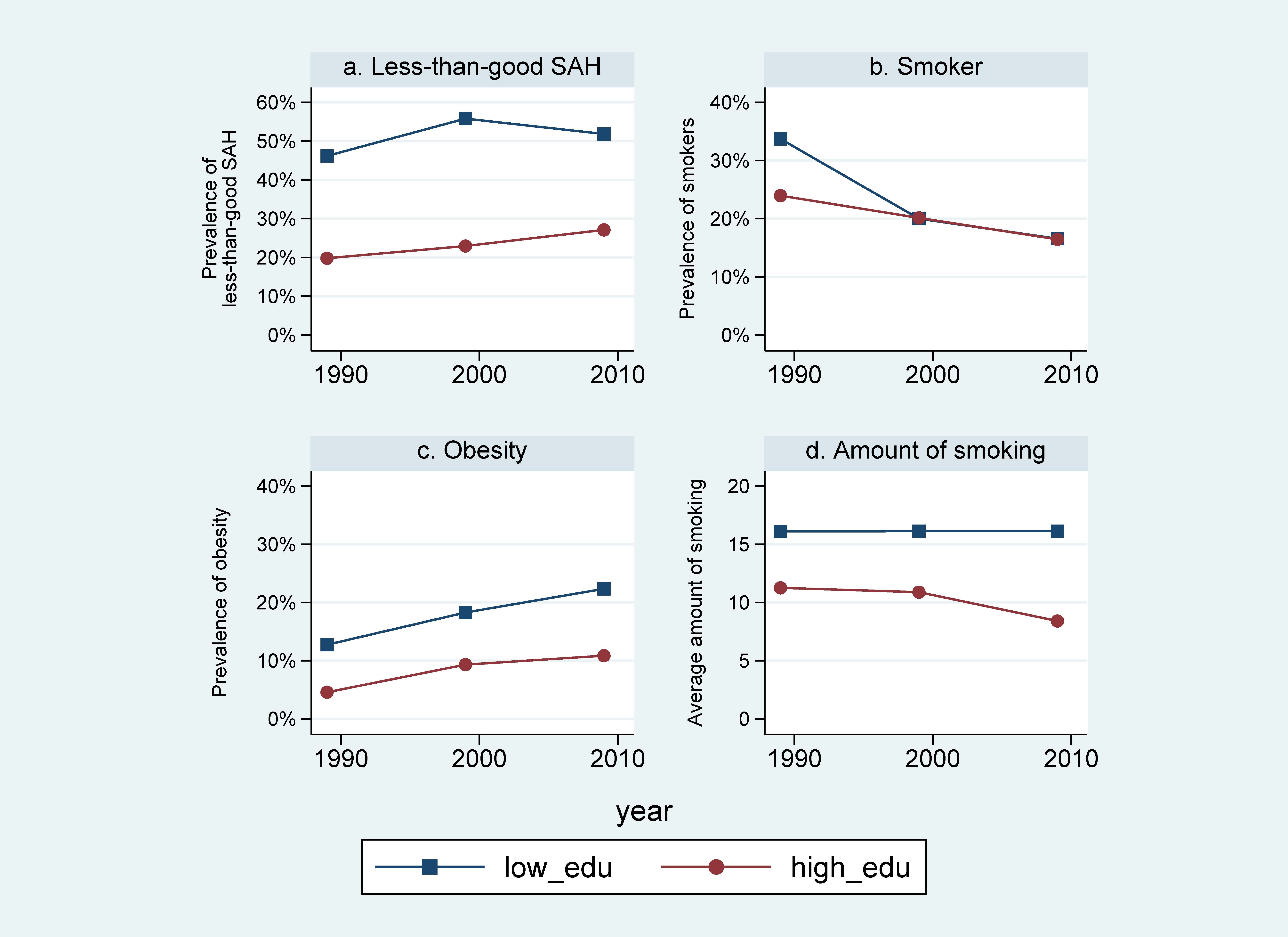

Supplement: Additional file 2: Figure SA1. — (JPG 886 kb) [file 12889_2016_3505_MOESM2_ESM.jpg]

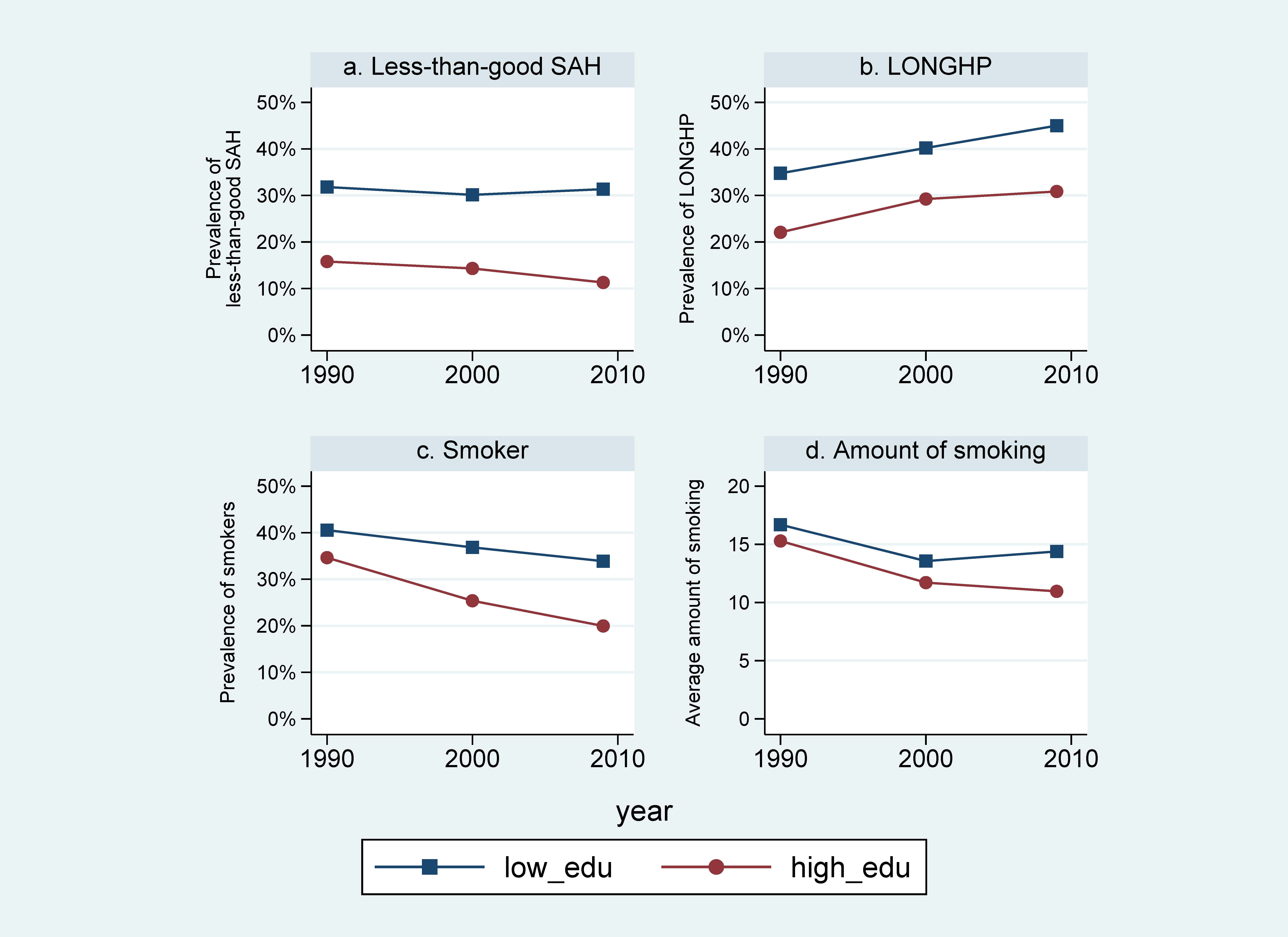

Supplement: Additional file 3: Figure SA2. — (JPG 891 kb) [file 12889_2016_3505_MOESM3_ESM.jpg]

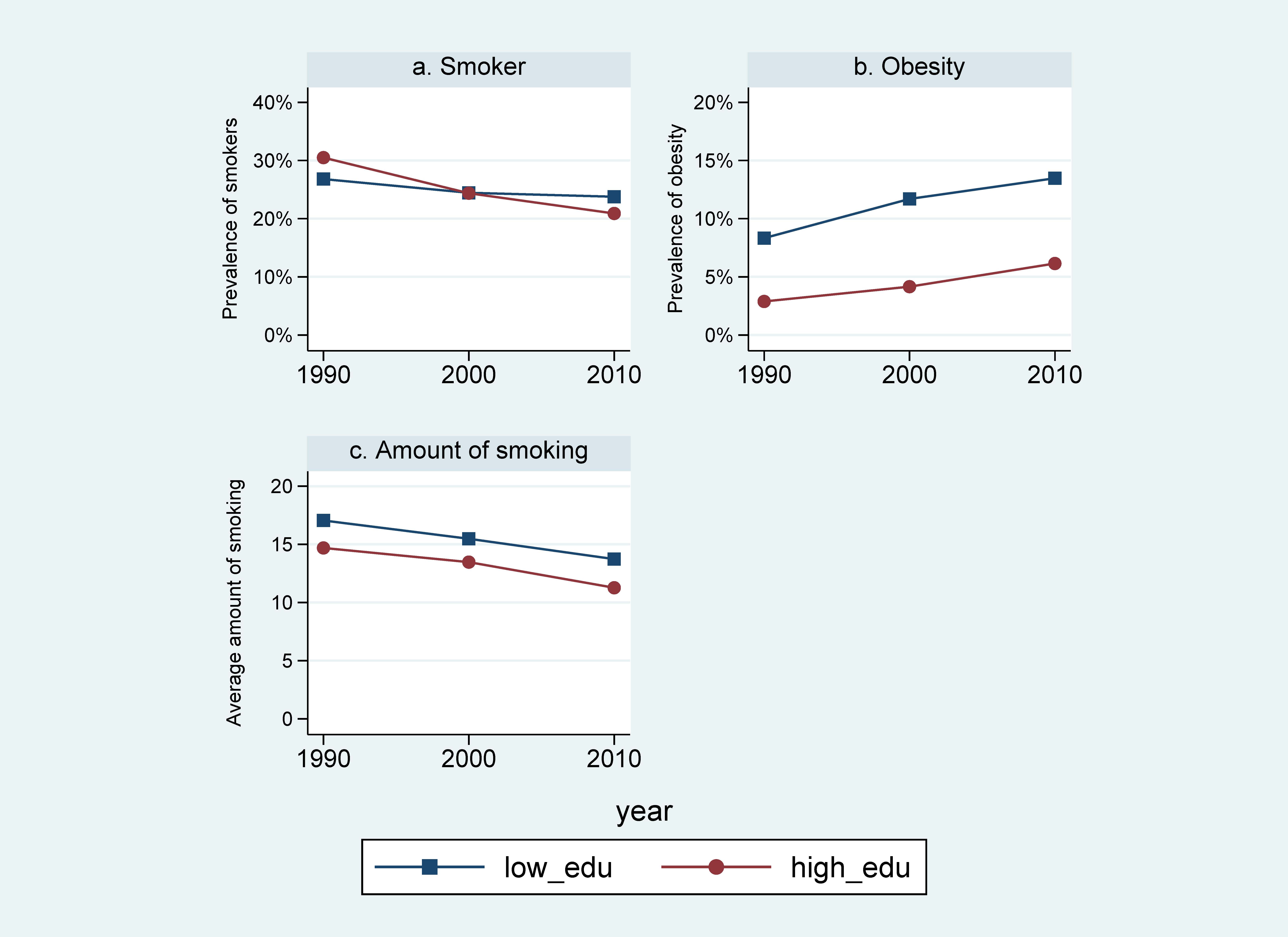

Supplement: Additional file 4: Figure SA3. — (JPG 775 kb) [file 12889_2016_3505_MOESM4_ESM.jpg]
